# A Tanshinone IIA Sulfonate Derivative Targets USP21 to Induce SCD1 Degradation, Suppressing NSCLC Metabolism and Enhancing Antitumor Immunity

**DOI:** 10.32604/or.2026.089127

**Published:** 2026-10-28

**Authors:** Yang Liu, Lixue Chen, Peng Wang, Xiaoli Ma, Shuaikang Pan, Dafu Yang, Tianyu Zhou, Fang Yu, Jincheng Song, Zhaolin Sun, Zhaoxia Dai, Peng Chu

**Affiliations:** 1 Department of Oncology, The Second Affiliated Hospital, Dalian Medical University, Dalian, China; 2 College of Pharmacy, Dalian Medical University, Dalian, China; 3 Institute of Cancer Stem Cell, Dalian Medical University, Dalian, China; 4 School of Pharmaceutical Engineering and Biotechnology, School of Cosmetics, Zhejiang Pharmaceutical University, No. 666 Siming Road, Ningbo, China; 5 College of Basic Medical Science, Dalian Medical University, Dalian, China

**Keywords:** Ubiquitin-specific peptidase 21, non-small cell lung cancer, stearoyl-CoA desaturase 1, tumor metabolism, tumor immunity

## Abstract

**Background:** The deubiquitinase ubiquitin-specific peptidase 21 (USP21) is a key regulator of tumor metabolism and immune homeostasis in non-small cell lung cancer (NSCLC). Its aberrant activation drives malignant growth, metabolic reprogramming and immune resistance, yet specific USP21 inhibitors for NSCLC therapy remain scarce. We aimed to develop a selective USP21 inhibitor, define its immunometabolic mechanism, and validate its anti-tumor and immunotherapy-sensitizing effects in NSCLC. **Methods:** We performed structure-based hierarchical virtual screening of a Food and Drug Administration (FDA)-approved drug library, followed by structure-guided optimization to generate USP21-in6. We characterized its USP21 inhibition, cytotoxicity and anti-tumor activity using *in* *vitro* NSCLC cell assays and *in vivo* syngeneic models. Lipidomic profiling and clinical sample analysis were also performed. **Results:** USP21-in6 potently and preferentially inhibits USP21 (half-maximal inhibitory concentration [IC50] = 0.61 μM; dissociation constant [Kd] = 1.3 μM). These quantitative parameters demonstrate the potent biochemical inhibitory activity of USP21-in6 toward USP21, supporting its suitability for subsequent cellular and *in-vivo* functional studies. This compound suppresses NSCLC proliferation and induces apoptosis, while exhibiting low toxicity toward normal cells. Mechanistically, USP21 stabilized stearoyl-CoA desaturase 1 (SCD1); USP21-in6 disrupted this USP21-SCD1 axis, triggering SCD1 degradation and blocking fatty acid synthesis. USP21-in6 monotherapy showed robust anti-tumor activity and boosted anti-programmed cell death protein 1 (PD-1) therapy by remodeling the tumor immune microenvironment: it enhanced cluster of differentiation 8-positive (CD8^+^) T-cell function and destabilized forkhead box protein 3 (Foxp3) to suppress regulatory T cells (Tregs). It lowered SCD1-derived monounsaturated fatty acids (C16:1, C18:1) in tumors and serum. Clinically, co-upregulated USP21 and SCD1 correlated with PD-1 inhibitor resistance. **Conclusion:** This work identifies USP21 as an important immunometabolic regulator in NSCLC. Targeting USP21 with USP21-in6 supports further investigation of USP21 inhibition as a potential dual strategy to overcome metabolic disorders and immune suppression for NSCLC immunometabolic therapy.

## 1 Introduction

Metabolic reprogramming constitutes a defining hallmark of malignant tumors, playing a pivotal driving role in tumor initiation, progression, and immune evasion [1,2,3]. Amongst these, the abnormal activation of fatty acid metabolism pathways stands out as a pivotal oncogenic mechanism. This pathway not only supplies energy and biosynthetic precursors for rapid tumor cell proliferation but also serves as a key molecular basis for tumor immune evasion by multidimensionally modulating the composition and function of the tumor immune microenvironment (TME) [4]. Even under conditions of abundant extracellular lipids, tumor cells remain highly dependent on endogenous *de novo* lipogenesis (DNL)—a shared metabolic phenotype across multiple malignancies, including lung, liver, and colorectal cancers [5]. The orderly progression of DNL is coordinated by the synergistic regulation of key rate-limiting enzymes: ATP citrate lyase (ACLY), fatty acid synthase (FASN), and stearoyl-CoA desaturase 1 (SCD1). Beyond their central roles in lipid synthesis, these enzymes perform complex and vital functions in tumor immune regulation, positioning them as core targets at the intersection of metabolism and immunity research [6,7].

Emerging evidence indicates that key DNL enzymes actively remodel the tumor immune microenvironment through diverse mechanisms. Elevated expression of certain enzymes suppresses anti-tumor immune cell infiltration and enhances the activity of immunosuppressive cells, while others elicit anti-tumor immunity by activating innate immune pathways and boosting effector T cell infiltration. Crucially, these functionalities exhibit marked tumor type dependency [8,9,10,11,12]. Concurrently, lipid metabolism dysregulation within the tumor microenvironment further exacerbates immunosuppression. For instance, lipid accumulation induces CD8^+^ T cell exhaustion, tumor-secreted lipids polarise tumor-associated macrophages towards the M2 phenotype, and the fatty acid metabolite prostaglandin E2 (PGE2) exerts dual pro-tumor proliferation and immunosuppressive effects [13,14,15]. Collectively, these findings position DNL enzymes as critical nodal points linking lipid metabolism and tumor immunity. Targeting these molecules to reverse immunosuppression represents a potential strategy for enhancing the efficacy of tumor immunotherapy, holding significant clinical relevance in lung cancer.

Non-small cell lung cancer (NSCLC) accounts for about 85% of all lung cancer cases and is the most commonly diagnosed and the deadliest cancer worldwide [16,17,18,19]. While smoking remains the primary risk factor, the proportion of cases occurring in never-smokers is rising, now accounting for roughly 10%–25% of the global burden, driven by air pollution, population aging, and indoor carcinogen exposure [20]. Prognosis for advanced-stage patients remains extremely poor, with stage IV, five-year survival rates below 10% [21,22,23]. Immune checkpoint inhibitors (ICIs) that block the PD-1/PD-L1 pathway have reshaped the clinical management of NSCLC, yet durable benefit remains confined to a minority of patients. A major reason is that profound immunosuppression within the tumor microenvironment drives both primary and acquired resistance [24]. Consequently, identifying upstream regulators of key DNL enzymes, elucidating core mechanisms of metabolic-immune cross-regulation, and exploring synergistic strategies combining metabolic interventions with ICIs represent pivotal directions for overcoming current therapeutic bottlenecks in NSCLC.

The ubiquitin-proteasome system (UPS) serves as a critical regulatory hub for maintaining cellular homeostasis. Deubiquitinating enzymes (DUBs) remove ubiquitin chains from substrate proteins, thereby counteracting ubiquitination. They are involved in both tumor development and immune regulation [25,26]. Abnormal expression or dysregulation of DUBs not only promotes malignant progression in tumor cells but also contributes to immune evasion by remodeling the tumor immune microenvironment, establishing them as potential core therapeutic targets [27]. Ubiquitin-specific protease 21 (USP21), one of the DUBs, stabilizes its substrate proteins and thereby regulates cell-cycle control and signal transduction [28,29,30,31]. USP21 exhibits significantly elevated expression in multiple malignancies [32,33,34,35,36], including NSCLC, with its levels correlating closely to tumor proliferation, invasion, metastasis, and poor patient prognosis [37,38,39,40]. In NSCLC, USP21 stabilizes PD-L1 protein to enhance immune evasion and sustains oncogenic signaling axes, including the EGFR/Lyn pathway, through YY1/SNHG16-mediated regulation, thereby establishing a pro-tumorigenic positive feedback loop [41,42,43,44]. Our previous work showed that inhibiting USP21 lowers FoxP3 levels in regulatory T cells (Tregs), which weakens their suppressive activity and strengthens antitumor immunity [45,46,47].

Recent studies have shown that DUBs contribute to tumor metabolic reprogramming by keeping key metabolic enzymes and transcription factors stable [48], suggesting that USP21 may also be linked to tumor fatty acid metabolism. However, whether USP21 regulates key DNL enzymes to influence NSCLC progression through the metabolism-immunity axis remains unexplored. Furthermore, although the pro-tumor and immunosuppressive roles of USP21 are well established, many existing USP21-targeting agents, including baicalin and PR-619, exhibit limited cellular potency and narrow therapeutic efficacy. While BAY-805 represents a potent and selective USP21 inhibitor, there remains a conspicuous lack of high-quality pharmacological tools suitable for in-depth biological and *in vivo* investigation [49,50,51,52].

Therefore, elucidating the functional mechanisms of USP21 in the metabolic-immune crosstalk of NSCLC, clarifying its regulatory role over key DNL enzymes, and developing specific inhibitors carry considerable theoretical and clinical translational value. We aimed to generate a novel small-molecule inhibitor with preferential USP21 activity, and investigate whether USP21 modulates tumor fatty acid synthesis and remodels the tumor immune microenvironment via regulating SCD1, thereby providing experimental evidence for immunometabolic targeted therapy in NSCLC.

## 2 Materials and Methods

### 2.1 Cell Lines and Culture Conditions

Human NSCLC cell lines (A549, H358, PC9, H1299, H460, H322), HEK293T, HBE, and murine LLC cells were obtained from the laboratory’s frozen stock. Short tandem repeat (STR) profiling was used to confirm the identity of all cell lines used in this study. A549, H322, HEK293T, and LLC were cultured in DMEM; others in RPMI-1640. Both media were supplemented with 10% fetal bovine serum (FBS) and 1% penicillin/streptomycin. Cells were maintained at 37°C with 5% CO_2_, tested for mycoplasma, and used within 20 passages.

### 2.2 Experimental Mice

Male C57BL/6J mice (6–8 weeks old, SPF grade) were purchased from Liaoning Changsheng Biotechnology Co., Ltd. Animals were maintained on a 12 h light/dark cycle (lights on from 8:00 a.m.) at 22 ± 2°C with 50–60% humidity, the animals had unrestricted access to regular chow and water. After 7 days of habituation, they were assigned to seven treatment groups (8 animals per group; 56 in total), with each animal constituting one experimental unit. No predefined animal inclusion or exclusion criteria were set, and every animal was retained in the final dataset. Investigators were not blinded during animal treatment, outcome assessment, or data analysis. Procedures were approved by the Institutional Animal Care and Use Committee of Dalian Medical University (Approval No. AEE24287) and followed the Guide for the Care and Use of Laboratory Animals.

### 2.3 Human Tissue Samples

A human NSCLC TMA (AF-LucSur2201, Aifang Bio) containing 80 NSCLC samples with matched adjacent non-cancerous tissues was used. The procurement and use of these samples complied with the Declaration of Helsinki and China’s Ethical Review Measures for Biomedical Research Involving Human Subjects. This study was approved by the Biomedical Science Ethics Review Committee of Hunan Fang Biotechnology Co., Ltd. (Ethical Approval No. HN20250401), and this approval covers all 80 paired tissue specimens in the TMA.

### 2.4 Virtual Screening and Lead Identification

The crystal structure of USP21 (PDB ID: 3MTN, https://www.rcsb.org/structure/3MTN) was downloaded from the RCSB-PDB database. Structure preparation and energy minimization were performed using GROMACS 2020.6, an open-source molecular dynamics package maintained by the GROMACS development team, Uppsala University, Uppsala, Sweden (https://www.gromacs.org). A library of 2662 FDA-approved drugs from DrugBank/ZINC20 (https://go.drugbank.com/ or https://zinc20.docking.org/) was docked to the active site (Cys221) using AutoDock Vina1.2.5 (The Scripps Research Institute, La Jolla, CA, USA). Key interactions were analyzed with PyMOL and Schrödinger Suite 2022 (Schrödinger, LLC, New York, NY, USA).

### 2.5 Lead Optimization via Single R-Group Substitution

A focused SAR study was performed by substituting the R-group on the phenyl ring of the initial hit. A library of 1088 R-groups was evaluated using Schrödinger Core Hopping. Promising analogs were further refined by FEP+ calculations, and those with ΔΔG_bind_ < −1.5 kcal/mol were selected for synthesis.

### 2.6 Molecular Dynamics (MD) Simulations

MD simulations started from the AutoDock Vina-predicted pose using GROMACS 2020.6 (Amber99sb). The topology and force-field parameters for small-molecule ligands were generated by Antechamber module within AmberTools. The generated parameters were converted to GROMACS-compatible format using ACPYPE. The Amber99sb-ILDN force field was applied for protein residues. The system was solvated with TIP3P water and neutralized with Na^+^/Cl^−^. After minimization and equilibration, a 100 ns NPT (1 atm, 300 K) run was performed. Binding free energy was calculated by MM/PBSA using 100 snapshots from the last 50 ns. The formula is as follows:

$$\begin{array}{l} \Delta Gbind\;=\;\Delta EMM\;+\;\Delta Gsolv\;\\ \Delta EMM\;=\;\Delta Eint\;+\;\Delta EvdW\;+\;\Delta Eele\\ \Delta Gsolv\;=\;\Delta GPB\;+\;\Delta GSA \end{array}$$

where Δ*Gbind*, binding free energy of protein-ligand complex; Δ*EMM*, gas-phase molecular-mechanics energy; Δ*Gsolv*, solvation free-energy contribution; *T*, absolute temperature; Δ*Eint*, internal bonded energy; Δ*EvdW*, van der Waals interaction energy; Δ*Eele*, electrostatic interaction energy; Δ*GPB*, polar solvation free energy from Poisson-Boltzmann calculation; Δ*GSA*, non-polar solvation free-energy term derived from solvent-accessible surface area.

### 2.7 Chemical Synthesis of USP21-in6

Under an Ar atmosphere, compound 2 (Tanshinone IIA Sulfonate, MCE, Monmouth Junction, NJ, USA), purity 98% by HPLC, catalog No. HY-N1370R, CAS No. 69659-80-9) was reacted with PCl5 (2.5 eq.) in POCl3 at 50°C for 6 h. Excess POCl3 was removed under vacuum by distillation. After cooling to room temperature, DCM/saturated NaHCO3 solution was added to the mixture. The organic layer was dried over anhydrous Na_2_SO_4_, then evaporated under vacuum. The obtained intermediate compound 3 was used directly for the next reaction without isolation, purification or yield determination. Compound 3 was then reacted with the amino substituent (3R,4R)-4-amino-2-methylpentane-1,5-diol (2.0 eq., synthesized in-house; no commercial CAS or catalog number available) and TEA (2.0 eq.) in a mixed solvent of DCM/DMF (1:1) and stirred at room temperature overnight. The reaction mixture was diluted with a large amount of water and extracted with DCM. The DCM layer was washed repeatedly with saturated brine, dried over anhydrous Na_2_SO_4_. The organic phase was concentrated under reduced pressure, and the resulting crude material was purified by flash chromatography on silica gel (DCM/MeOH, 100:1 → 50:1), affording compound 1 (USP21-in6) as a red solid (461.55 g/mol).

Compound 1 (Red solid), Molecular weight: 461.55. ^1^H and ^13^C NMR spectra were recorded on a Bruker Avance 400 spectrometer operating at 400.15 MHz for ^1^H and 100.63 MHz for ^13^C. All experiments were performed at 298.0 K using DMSO-d_6_ as solvent. Spectra were acquired with standard Bruker pulse programs (zg30 for ^1^H, zgpg30 for ^13^C). Chemical shifts (δ) values are given in ppm and referenced to the residual DMSO-d_6_ signal. ^1^H NMR (400 MHz, DMSO-d6) δ7.99 (1H, d, J = 8.31 Hz), 7.88 (1H, d, J = 8.13 Hz), 7.64 (1H, d, J = 8.13 Hz), 4.52 (2H, t, J = 5.20 Hz), 3.87–3.77 (1H, m), 3.50–3.45 (1H, m), 3.29–3.24 (1H, m), 3.10 (2H, t, J = 6.24 Hz), 2.43 (3H, s), 1.78–1.70 (2H, m), 1.65–1.60 (2H, m), 1.30 (6H, s), 0.97 (3H, d, J = 6.37 Hz). ^13^C NMR (101 MHz, DMSO-d_6_) δ181.9, 175.3, 160.4, 151.1, 147.1, 143.5, 133.9, 128.0, 126.0, 125.1, 120.9, 119.9, 64.9, 61.1, 60.8, 37.8, 35.0, 31.9 (2C), 30.0, 19.8, 19.2, 9.8.

The identity of USP21-in6 was confirmed by ^1^H and ^13^C NMR spectroscopy. This study focuses on the biological function of USP21-in6, rather than synthetic methodology.

### 2.8 Free Energy Landscape (FEL) Analysis

Molecular dynamics (MD) simulations were performed using GROMACS 2020.6 following the standard protocol recommended for the Amber99sb-ILDN force field (http://www.gromacs.org; Amber reference manual). Ligand topologies were generated with Antechamber (AmberTools) and converted with ACPYPE. Each system was solvated with TIP3P water and neutralized with Na^+^/Cl^−^. Following energy minimization and sequential NVT/NPT equilibration with position restraints on the heavy atoms of the complex, production simulations were performed in the NPT ensemble at 300 K and 1 bar using the default temperature- and pressure-coupling schemes of the standard protocol, a 2 fs integration time step with bond constraints on hydrogen atoms, a 1.0 nm cut-off for short-range interactions, and the particle mesh Ewald (PME) method for long-range electrostatics. The USP21–USP21-in6 complex was simulated for 100 ns, and each candidate complex for 10 ns (single production run per system). For trajectory analysis, protein-backbone root mean square deviation (RMSD) and radius of gyration (Rg) were calculated using the gmx rms and gmx gyrate modules of GROMACS, respectively. For ligand RMSD analysis, all heavy atoms of each ligand were selected (hydrogen atoms excluded), the energy-minimized initial ligand conformation served as the reference structure, and each trajectory frame was fitted to the protein-backbone atoms to remove global translational and rotational motions; ligand RMSD was analysed across the full 10 ns screening trajectory at 100 ps frame intervals and used as a criterion to identify compounds with stable binding in the USP21 pocket. The Gibbs free energy landscape was constructed using the gmx sham tool based on the 50 ns trajectory, with protein-backbone RMSD and Rg as reaction coordinates, and the resulting 2D free-energy maps were visualized as 3D surface plots using custom Python scripts.

### 2.9 Ubiquitin-Rhodamine 110 (Ub-Rho110) Assay

Deubiquitinase (DUB) activity was measured using a fluorogenic substrate, Ub-Rho110, as previously described [53]. The reaction buffer contained 50 mM Tris-HCl (pH 7.5), 0.01% (v/v) Triton X-100, 2.5 mM DTT, and 0.1% (w/v) bovine gamma globulin. Information of the full-length recombinant human proteins used in the experiment is provided in Table S1. Reactions were performed in black 384-well plates (PerkinElmer, Cat# 6008260, Waltham, MA, USA) with a total volume of 20 μL. Each well contained 600 nM enzyme and serially diluted compounds, and the mixture was pre-incubated at room temperature for 1 h to allow inhibitor-enzyme binding. The reaction was then initiated by adding Ub-Rho110 substrate to a final concentration of 3 μM (Boston Biochem, Cat# U-555-050, Cambridge, MA, USA). Fluorescence intensity (excitation/emission: 485/535 nm) was immediately measured using a Varioskan LUX microplate reader (Thermo Fisher Scientific, Waltham, MA, USA) within the linear phase of product generation to avoid substantial substrate depletion. Nonlinear curve fitting in GraphPad Prism 10 yielded the IC50 values. (GraphPad Software, Boston, MA, USA).

### 2.10 Bio-Layer Interferometry (BLI) Assay

Binding kinetics/affinity were determined via Octet R8 BLI (ForteBio, Fremont, CA, USA). Recombinant USP21 (Boston Biochem, Cat# E-622, Cambridge, MA, USA) was biotinylated with EZ-Link™ NHS-PEG4-Biotin (Thermo Fisher, Cat# 21329, Waltham, MA, USA) for 1 h at RT; unreacted biotin was removed by a Zeba™ Spin Desalting Column, 7K MWCO (Thermo Fisher Scientific, Cat# 89889, Waltham, MA, USA). Biotinylated USP21 was immobilized on SSA biosensors (ForteBio, Cat# 18-5057, Fremont, CA, USA). To eliminate bulk refractive index mismatch and solvent jump artifacts, the BLI kinetics buffer (PBS, pH 7.4, 0.01% BSA, 0.002% Tween-20) was supplemented with an equal percentage of DMSO to match the vehicle concentration of compound working solutions. After baseline stabilization in kinetics buffer, sensors were exposed to serially diluted compounds (association, 60 s) and buffer-only wells (dissociation, 60 s) at 30°C/1000 rpm. Binding curves were globally fitted to a 1:1 model using Octet BLI Software 12.2 (Sartorius, Fremont, CA, USA) to obtain k_on, k_off, and K_D.

### 2.11 Cellular Thermal Shift Assay (CETSA)

USP21-in6 was dissolved in DMSO as a stock solution; in all cell-based assays the final DMSO concentration was kept below 0.1% (v/v), with vehicle-matched controls included. H1299 or H460 cells were exposed to 25 μM USP21-in6 or DMSO for 3 h at 37°C, harvested by trypsinization, rinsed once with 1× phosphate-buffered saline (PBS, pH 7.4), and adjusted to 2 × 10^7^ cells/mL. 50 μL aliquots were heated in a thermal cycler (T100, Bio-Rad Laboratories, Hercules, CA, USA) at 37–55°C (3 min gradient), followed by 3 min at RT and snap-freezing on ice. Cells were lysed via three liquid nitrogen freeze-thaw cycles in detergent-free 1× PBS (pH 7.4) to preserve the separation of soluble and heat-aggregated protein fractions. Lysates were centrifuged (20,000× *g*, 20 min, 4°C); supernatants were mixed with 2× SDS loading buffer (Cat. No. P0015B, Beyotime, Shanghai, China), boiled, and analyzed for USP21 by Western blot. Band intensities were quantified with ImageJ (version 1.54f, National Institutes of Health, Bethesda, MD, USA), and soluble protein fraction was plotted against temperature.

### 2.12 In Vitro Deubiquitination Assay (Di-Ub Assay)

Purified recombinant USP21 protein (amino acids 196-565) at a final concentration of 0.2 μM was incubated with 2 μg of K48-linked Di-Ubiquitin (Di-Ub, Cat. No. P20022, Solarbio, Beijing, China) in reaction buffer (20 mM HEPES (Cat. No. ST080, Beyotime, Shanghai, China), pH 7.2, 150 mM NaCl (Cat. No. ST1229, Beyotime, Shanghai, China), 10 mM dithiothreitol (DTT, Cat. No. ST041, Beyotime, Shanghai, China)) at 37°C for 3 h. USP21-in6 or DMSO was added to the reaction mixture. Adding 2× SDS loading buffer and boiling stopped the reaction. The products were then separated on SDS-PAGE gels and probed for ubiquitin by immunoblotting.

### 2.13 Cell Viability Assay (CCK-8)

Logarithmic-phase cells (A549, H1299, H460, H358, PC9, H322, HEK293T and HBE) were seeded into 96-well plates at 5 × 10^3^ cells/well. After overnight incubation, cells were treated with USP21-in6 at serial concentrations of 0, 0.5, 1, 2, 5, and 10 μM for 24 h. CCK-8 reagent (Cat. No. MA0218, Meilunbio, Dalian Meilun Biotechnology Co., Ltd., Dalian, China) was added to each well to a final concentration of approximately 10% (v/v). The plates were then kept at 37°C for 2–4 h before reading absorbance at 450 nm on a microplate reader (Varioskan LUX, Thermo Fisher Scientific, Waltham, MA, USA). Viability was expressed relative to the DMSO-treated wells, and IC_50_ values were obtained from nonlinear regression in GraphPad Prism (GraphPad Software, Boston, MA, USA).

### 2.14 Clonogenic Assay

H1299 or H460 cells was seeded at low density (1000 cells/well) in 6-well plates and treated with USP21-in6 after 24 h. Medium was refreshed every 3–4 days. Fresh USP21-in6 was supplemented each time when the medium was replaced to maintain consistent drug concentration throughout the incubation period. After 10–14 days, colonies were fixed with 4% paraformaldehyde (PFA, Cat. No. P0099, Beyotime, Shanghai, China). and stained with 0.1% crystal violet (Cat. No. C0121-100 mL, Beyotime, Shanghai, China). ImageJ (version 1.54f, National Institutes of Health, Bethesda, MD, USA) was used to count colonies containing more than 50 cells.

### 2.15 Wound Healing (Scratch) Assay

For the scratch assay, confluent monolayers of H1299 and H460 cells in six-well plates (3 × 10^5^ cells per well) were wounded once with a sterile 200-μL pipette tip. Following a rinse with 1× PBS (pH 7.4) to clear detached cells, the monolayers were maintained in 0.5% FBS medium containing the indicated concentrations of USP21-in6. Bright-field images of the same fields were taken immediately and at 24 and 48 h post-wounding. Gap widths were quantified at several positions with ImageJ (version 1.54f, National Institutes of Health, Bethesda, MD, USA), from which the relative migration rate was derived.

### 2.16 Transwell Invasion Assay

Cell invasion was evaluated using Matrigel-coated Transwell chambers (8.0-μm pore; Cat. No. 3422, Corning Incorporated, Corning, NY, USA). The upper membrane was layered with 60 μL of Matrigel basement membrane matrix (Cat. No. 082704, Mogengel Biotech, Xiamen, China) at a 1:8 dilution in serum-free medium and solidified at 37°C for 2 h. H1299 or H460 cells (5 × 10^4^) in 200 μL of serum-free RPMI 1640 medium (Cat. No. MA0215, Meilunbio, Dalian, China) containing USP21-in6 were placed in the upper chamber, while the lower chamber received 600 μL of 10% FBS RPMI 1640 medium as a chemoattractant. Once the 24 h incubation ended, we removed the cells still sitting on the top side of the membrane using cotton swabs. Those that had crossed over were fixed in 4% paraformaldehyde (PFA; Cat. No. P0099, Beyotime, Shanghai, China), followed by staining with 0.1% crystal violet (Cat. No. C0121-100 mL, Beyotime, Shanghai, China); five random fields of each well were then counted under an inverted microscope (Eclipse Ti2-U, Nikon, Tokyo, Japan).

### 2.17 Plasmid Transformation, Amplification, and Extraction

Plasmid transformation, amplification, and extraction were performed as follows: 1 μL plasmid (MiaoLing Biology, Wuhan, China). was mixed with 50 μL competent E. coli DH5α (Cat. No. CD201-01, TransGen Biotech, Beijing, China) on ice for 30 min, heat-shocked at 42°C for 45 s, chilled on ice for 2 min, and recovered in 1 mL LB broth (37°C, 180 rpm) for 1 h before being spread on antibiotic-containing LB agar plates (ampicillin, Cat. No. MA0110, Meilunbio, Dalian, China at 50 μg/mL or kanamycin sulfate, Cat. No. MA0120, Meilunbio, Dalian, China at 50 μg/mL) and incubated overnight at 37°C; a single colony was then inoculated into 5 mL antibiotic-supplemented LB broth and cultured overnight at 37°C (220 rpm); plasmid DNA was extracted with Plasmid Extraction Kit (Cat. No. D6942-01, Omega Bio-tek, Norcross, GA, USA) per manufacturer’s instructions, involving resuspension, lysis, and neutralization of bacterial pellets, centrifugation at 12,000× *g* for 1 min to clear lysates, application of supernatants to HiBind^®^ DNA columns, elution of plasmid DNA with nuclease-free water (Cat. No. MA0080, Meilunbio, Dalian, China) after washing, and determination of concentration and purity via Nanodrop (Nanodrop One, Thermo Fisher Scientific, Waltham, MA, USA).

### 2.18 Cell Transfection

For transfection, HEK293T, H1299 and H460 cells were plated in 6-well plates at 2 × 10^5^ per well and grown until 60–70% confluent, at which point Lipofectamine 2000 (Invitrogen) was introduced following the supplier’s instructions. For each well of a 6-well plate, a mixture of 2.5 μg plasmid DNA in 125 μL Opti-MEM (Cat. No. 11668019, Invitrogen, Waltham, MA, USA) was prepared. In a separate tube, 7.5 μL Lipofectamine 2000 was diluted in 125 μL Opti-MEM. The two solutions were combined, incubated for 15 min at room temperature, and then added dropwise to the cells. Medium was changed 6 h post-transfection, and cells were harvested 24–48 h later. As for siRNA transfection, siRNA duplexes used in this study were purchased from MiaoLing Biology, Wuhan, China. Transfections were performed with siRNA at 50 nM (100 pmol per well of 6-well plates). After a 20-min incubation at room temperature, the siRNA-Lipofectamine 2000 complexes were applied to the cells, which were then cultured for 48 h before further experiments. For stable knockdown, lentiviral particles encoding USP21-specific shRNA or scrambled control shRNA were used. Cells were seeded at a density of 1.5 × 10^5^ cells per well in 6-well plates and infected with lentivirus in the presence of 8 μg/mL polybrene (Cat. No. MB2481, Meilunbio, Dalian, China). Puromycin (Cat. No. MB2771, Meilunbio, Dalian, China) was added at 2 μg/mL 48 h post-infection, and the cells were passaged under this selection for a minimum of one week. Knockdown efficiency was confirmed by Western blot. Lentiviruses and plasmids are provided in Table S2.

### 2.19 Quantitative Real-Time PCR (qRT-PCR)

RNA was purified from H1299 and H460 cells with the FastPure Cell/Tissue Total RNA Isolation Kit (Cat. No. RC112-01, Vazyme, Nanjing, China), and its concentration and quality were determined on a NanoDrop (Nanodrop One, Thermo Fisher Scientific, Waltham, MA, USA). One microgram of total RNA was then converted to cDNA using HiScript^®^ II Q RT SuperMix for qPCR (+gDNA wiper) (Cat. No. R223-01, Vazyme, Nanjing, China); the resulting cDNA was diluted with nuclease-free water to minimize carry-over of PCR inhibitors from the reverse-transcription buffer. Real-time amplification used ChamQ Universal SYBR qPCR Master Mix (Cat. No. Q711-02, Vazyme, Nanjing, China), run on a CFX96 Real-Time PCR Detection System (Bio-Rad Laboratories, Hercules, CA, USA). Per 20-μL reaction, the mixture included 10 μL of the Master Mix, 0.4 μL apiece of the forward and reverse primers (10 μM), 2 μL of cDNA, and 7.2 μL nuclease-free water. Thermocycling began with 30 s at 95°C, then continued for 40 repeats of 10 s at 95°C and 30 s at 60°C. Relative expression was calculated against GAPDH using 2^−ΔΔCt^. The primers used are given in Table S3.

### 2.20 Western Blotting

HEK293T, H1299 and H460 cells were homogenized in RIPA lysis buffer (Cat. No. P0013D, Beyotime, Shanghai, China) supplemented with 1 mM PMSF (Cat. No. ST506, Beyotime, Shanghai, China) and a protease inhibitor cocktail (Cat. No. P1006, Beyotime, Shanghai, China), and the lysates were cleared by centrifugation at 14,000× *g* for 15 min at 4°C. Protein concentrations were measured with the BCA Protein Assay Kit (Beyotime) against a BSA standard series (0–2.0 mg/mL). Equal amounts of protein (20–40 μg) were resolved on 8–12% SDS-PAGE gels and electrotransferred to PVDF membranes (Amersham Hybond P 0.2, Cat. No. 10600021, Cytiva, Marlborough, MA, USA). After blocking for 1 h at room temperature in TBST (Tris-buffered saline with 0.1% Tween-20) containing 5% non-fat milk (Cat. No. D8340, Solarbio, Beijing, China), the membranes were probed with primary antibodies overnight at 4°C, washed in TBST, and then incubated with HRP-conjugated secondary antibodies for 1–2 h at room temperature. Protein bands were detected by enhanced chemiluminescence (ECL) and captured on an Amersham Imager 600 (Cytiva). Band intensities were quantified with ImageJ (v1.54f, NIH). Antibodies are listed in Table S4.

### 2.21 Co-Immunoprecipitation (Co-IP)

HEK293T, H1299 and H460 cells were lysed in IP lysis buffer (25 mM Tris-HCl pH 7.4, 150 mM NaCl, 1% NP-40, 1 mM EDTA, 5% glycerol) containing protease inhibitors (Cat. No. P1006, Beyotime, Shanghai, China). Cell debris was removed by centrifugation at 14,000× *g* for 15 min at 4°C. For pre-clearing, 1 μg of control IgG (Cat. No. #2975, Cell Signaling Technology, Danvers, MA, USA) and 20 μL of Protein A/G Agarose beads (Cat. No. 17-0618-01, Cytiva, Marlborough, MA, USA) were added to the lysate and incubated for 1 h at 4°C. The pre-cleared supernatant was then incubated with the specific primary antibody (2–5 μg) overnight at 4°C with rotation. Protein A/G Agarose beads were added and incubated for an additional 4 h. The beads were then rinsed five times in ice-cold IP buffer. Captured proteins were released by boiling in 2× SDS loading buffer (Cat. No. P0015B, Beyotime, Shanghai, China) for 5 min and subsequently examined by immunoblotting.

### 2.22 Protein Stability Assay

Cells were treated with 20 μg/mL cycloheximide (CHX, Cat. No. U1013, Univ-bio, Shanghai, China) to inhibit new protein synthesis. Cells were harvested at indicated time points (0, 1, 2, 4, 6, 8 h) post-CHX treatment. Total protein was extracted and analyzed by Western blotting for the target protein. Band signals were analyzed with ImageJ (version 1.54f, National Institutes of Health, Bethesda, MD, USA) and expressed relative to the loading control. Protein half-life was estimated by plotting the relative protein level against time.

### 2.23 Free Fatty Acid (FFA) Content Assay

Cellular FFA content was measured using a Free Fatty Acid Assay Kit (Cat. No. BC0595, Solarbio, Beijing, China) according to the manufacturer’s instructions. Briefly, cells were homogenized, and lipids were extracted. The extracted FFA was reacted with a copper reagent (Cat. No. BC0595, Solarbio, Beijing, China) to form a colored complex, the absorbance of which was measured at 550 nm using a Multimode Microplate Reader (Varioskan LUX, Thermo Scientific, Waltham, MA, USA). A standard curve was prepared using palmitic acid standards, and FFA content was normalized to total protein concentration.

### 2.24 Oil Red O Staining

Cells were fixed with 4% paraformaldehyde (PFA, Cat. No. P8045, Solarbio, Beijing, China) for 20 min, washed with 1× PBS (PH 7.4), and incubated with 60% isopropanol (diluted from pure isopropanol, Cat. No. I8290, Solarbio, Beijing, China) for 5 min. After staining with fresh Oil Red O (0.3% in 60% isopropanol) for 15 min, excess dye was removed with 60% isopropanol and water. Nuclei were counterstained with hematoxylin (Cat. No. H8070, Solarbio, Beijing, China) for 1–2 min, and images were captured using an inverted microscope (Eclipse Ti2-U, Nikon, Tokyo, Japan). The dye was then extracted from the stained cells with 100% isopropanol (Cat. No. I8290, Solarbio, Beijing, China), and the absorbance of the extract read at 520 nm on a Multimode Microplate Reader (Varioskan LUX, Thermo Scientific, Waltham, MA, USA).

### 2.25 Transmission Electron Microscopy (TEM)

H1299 and H460 cells were fixed with 2.5% glutaraldehyde (diluted from glutaraldehyde stock solution, Cat. No. G8055, Solarbio, Beijing, China) in 0.1 M phosphate buffer (pH 7.4, self-prepared using sodium dihydrogen phosphate (Cat. No. S8180, Solarbio, Beijing, China) and disodium hydrogen phosphate (Cat. No. S8232, Solarbio, Beijing, China)) at 4°C overnight, washed, and post-fixed with 1% osmium tetroxide (diluted from 4% osmium tetroxide stock solution, Cat. No. 19100, Electron Microscopy Sciences, Hatfield, PA, USA) for 2 h. After dehydration in a graded ethanol series (30–100%), cells were embedded in epoxy resin. Ultrathin sections (70 nm) were cut using an ultramicrotome (EM UC7, Leica, Wetzlar, Germany), stained with uranyl acetate (Cat. No. 22400, Electron Microscopy Sciences, Hatfield, PA, USA) and lead citrate (Cat. No. 17800, Electron Microscopy Sciences, Hatfield, PA, USA), and examined under a Hitachi HT7800 transmission electron microscope (Hitachi, Tokyo, Japan) at 80 kV.

### 2.26 Flow Cytometry for Lipid Droplets

Following harvest and a rinse in 1× PBS (pH 7.4), the cells were incubated with BODIPY™ 493/503 (Cat. No. D3922, Invitrogen, Waltham, MA, USA; 1:1000 dilution in PBS) for 15 min at room temperature, protected from light. Two further rinses in FACS buffer (PBS with 2% FBS) followed, after which the cells were taken up in fresh buffer for analysis. Fluorescence intensity was analyzed immediately using flow cytometer (Thermo Fisher Scientific, Sony MA900, Waltham, MA, USA). Data were processed with FlowJo V10 software (Version 10, FlowJo, LLC, Ashland, OR, USA).

### 2.27 Hematoxylin and Eosin (H&E) Staining

Specimens were fixed in 10% neutral buffered formalin (Cat. No. G2161, Solarbio, Beijing, China), embedded in paraffin, and cut into 4-μm sections. For staining, sections were first dewaxed in xylene (Cat. No. X8390, Solarbio, Beijing, China) and rehydrated through graded alcohols; nuclei were then stained with Mayer’s hematoxylin (Cat. No. G1080, Solarbio, Beijing, China) for 5 min, differentiated in 1% acid alcohol (prepared in-house), and blued in 0.5% sodium bicarbonate (prepared from sodium bicarbonate powder, Cat. No. S8230, Solarbio, Beijing, China). Cytoplasm was counterstained with eosin (Cat. No. G1100, Solarbio, Beijing, China) for 1 min. Following dehydration and a final xylene rinse, the sections were coverslipped with neutral resin and photographed under a Nikon Eclipse Ci-L microscope (Nikon, Tokyo, Japan).

### 2.28 Immunohistochemistry (IHC)

Paraffin sections were first dewaxed and rehydrated, then subjected to antigen retrieval in citrate (pH 6.0, Cat. No. C1032, Solarbio, Beijing, China) or EDTA (pH 9.0) buffer using microwave heating (20 min). To quench endogenous peroxidase, sections were treated with 3% H_2_O_2_ (Cat. No. H1125, Solarbio, Beijing, China) for 15 min, and non-specific binding was then reduced by a 30-min incubation in 10% goat serum (prepared from goat serum, Cat. No. SL038, Solarbio, Beijing, China). Primary antibody incubation was carried out overnight at 4°C, followed by a 1-h application of the HRP-conjugated secondary antibody at ambient temperature; signal was developed with DAB and nuclei counterstained with hematoxylin. Slides were digitized on a Panoramic MIDI scanner (3DHISTECH) and evaluated with ImageJ.

### 2.29 In Vivo Mouse Studies

Fifty-six C57BL/6J mice were randomly divided into 7 groups (n = 8). LLC cells (1 × 10^6^) in 100 μL PBS were injected subcutaneously into the right flank. When tumors reached an average volume of approximately 100 mm^3^ (about 8 × 5 mm), treatment was initiated as follows: (1) Vehicle control (saline with 5% DMSO, 5% Tween-80, 90% saline); (2) USP21-in6 low dose (10 mg/kg, i.p., daily); (3) USP21-in6 high dose (20 mg/kg, i.p., daily); (4) SCD1 inhibitor (SCD1-in, A939572, 10 mg/kg, i.p., daily); (5) Anti-PD-1 antibody (5 mg/kg, i.p., twice weekly); (6) Combination: USP21-in6 (10 mg/kg) + A939572 (10 mg/kg); (7) Combination: USP21-in6 (10 mg/kg) + Anti-PD-1 (5 mg/kg, twice weekly). Tumor dimensions were measured every other day with calipers, and volume was calculated as (Length × Width^2^)/2. Body weight was monitored simultaneously. The treatment lasted for 20 days. On day 21, mice were anesthetized with an intraperitoneal injection of 2.5% tribromoethanol (10 μL/g). Blood was collected via retro-orbital puncture. Blood samples were spun at 3000× *g* for 15 min at 4°C to collect the serum, which was kept at −80°C until use. The mice were then sacrificed by cervical dislocation. Tumors, spleen, lymph nodes (inguinal, axillary, cervical), heart, liver, lungs, and kidneys were harvested, weighed, and processed for further analysis.

### 2.30 Flow Cytometric Analysis of Immune Cells

Single-cell suspensions of tumors, spleens, and lymph nodes were generated as follows: tumor tissue was chopped and enzymatically dissociated in Collagenase IV (4 mg/mL, Worthington) plus DNase I (0.1 mg/mL, Solarbio) at 37°C for 30 min, passed through a 70-μm strainer, and treated with ACK lysis buffer to eliminate erythrocytes. For intracellular cytokine measurements, aliquots of the suspension were re-stimulated *ex vivo* with PMA (Cat. No. P8139, Sigma-Aldrich, St. Louis, MO, USA) and ionomycin (Cat. No. I0634, Sigma-Aldrich, St. Louis, MO, USA) plus Brefeldin A (Cat. No. 420601, BioLegend, San Diego, CA, USA) for 4 h at 37°C before staining. Surface staining was preceded by an Fc-block step (anti-CD16/32) to minimize non-specific binding; the cells were then labelled with fluorochrome-conjugated antibodies against CD45, CD4, and CD8 at 4°C for 30 min. Intranuclear staining of FoxP3 and intracellular staining of Granzyme B and IFN-γ required fixation and permeabilization with the BD Transcription Factor Buffer Set. All samples were acquired and sorted on a Sony MA900 cell sorter (Sony Biotechnology, San Jose, CA, USA), and the data were processed in FlowJo V10.

### 2.31 Targeted Lipidomics by GC-MS

Approximately 50 mg snap-frozen tumor tissue or 100 μL mouse serum was extracted with 1 mL dichloromethane: methanol (1:1, v/v), vortexed, sonicated on ice for 15 min, and centrifuged at 13,000× *g* for 10 min at 4°C. The supernatant (500 μL) was dried under nitrogen, resuspended in 0.3 M methanolic KOH, and incubated at 60°C for 30 min for transesterification. Once the mixture had cooled, 0.5 mL hexane was layered on, and the upper phase containing fatty acid methyl esters (FAMEs) was recovered for GC-MS work. Separations were achieved on an Agilent 8890 GC system coupled to a 7000 D triple quadrupole mass spectrometer, fitted with a DB-FastFAME column (20 m × 0.18 mm × 0.2 μm) and using helium (1.0 mL/min) as the carrier gas. Injection was performed at 230°C, with 1 μL of each sample introduced in split mode (50:1). The column temperature started at 80°C for 0.5 min, then rose to 175°C at 70°C/min and finally to 230°C at 8°C/min, where it was maintained for 1 min. Mass detection used EI mode (70 eV) with selected ion monitoring (SIM); quantification was handled in Agilent MassHunter Quantitative Analysis software (v10.0). Targeted GC-MS quantification of medium- and long-chain fatty acids was performed using external-standard calibration without isotope-labeled internal standards, with quantification referenced to mixed fatty-acid methyl-ester external standards. Prior to GC-MS injection, all samples were randomized to minimize systematic batch bias. The S4-level mixed standard solution was employed as quality-control (QC) sample, and one QC sample was injected for every 5–10 test samples throughout data acquisition. Instrument performance was considered acceptable when QC peak-area relative standard deviation (RSD) was less than 10%; in the present study, peak-area RSD values for all 36 fatty-acid methyl esters were below 8.04%. The calibration range was 0.099–263 μg/mL for palmitic acid methyl ester (C16:0) and 0.049–131.5 μg/mL for the other 35 analytes, and all calibration curves achieved coefficients of determination (R^2^) ≥0.996. Analytes were identified by matching GC retention time and characteristic mass-spectrometric fragment ions to authentic mixed standards under selected-ion monitoring (SIM) mode. Peak integration was automatically completed in MassHunter software (v10.0.707.0), followed by manual visual verification. Of note, formal extraction-recovery assessment was not conducted by the service provider for this batch of samples, which represents a limitation of this assay.

### 2.32 Clinical Samples

Clinical samples were collected from the Second Affiliated Hospital of Dalian Medical University. Eligibility criteria were as follows: histologically confirmed NSCLC, age 18–70 years, Eastern Cooperative Oncology Group (ECOG) performance status of 0–2, and treatment with anti-PD-1 monotherapy (pembrolizumab, nivolumab, sintilimab, camrelizumab, tislelizumab, or penpulimab) at standard recommended doses in accordance with clinical guidelines. Pretreatment sections were retrieved from archived specimens in the Department of Pathology. Response evaluation followed the Response Evaluation Criteria in Solid Tumors version 1.1 (RECIST 1.1): contrast-enhanced chest and abdominal CT scans were performed after two cycles of treatment and confirmed at the 6-month follow-up. Patients achieving complete or partial response were classified as responders, whereas those with stable or progressive disease were grouped as non-responders. The study protocol was approved by the Ethics Committee of the Second Affiliated Hospital of Dalian Medical University (approval No. KY2025-724-01-01), and written informed consent was obtained from all patients before sample collection.

### 2.33 Multiplex Immunofluorescence (mIF) Staining

MIF staining was performed on human NSCLC sections using the Opal™ 7-Color Manual IHC Kit (Cat. No. NEL811001KT, Akoya Biosciences, Marlborough, MA, USA) based on TSA technology. USP21, SCD1, CD8, PD-L1, and panCK were stained sequentially: once retrieval and blocking were done, each primary antibody was applied overnight at 4°C; the sections were then treated with the corresponding HRP-conjugated secondary antibody, after which the fluorophore-conjugated TSA Opal dye was added. Antibody complexes were stripped by microwave heating between rounds. Nuclei were counterstained with DAPI, and slides were mounted with anti-fade medium. Images were acquired using a Vectra Polaris system (Akoya Biosciences) and analyzed with inForm^®^ software. The detailed information of the reagents and instruments used in this study is listed in Tables S5 and S6.

### 2.34 Gene Set Variation Analysis (GSVA)

Single-sample gene set enrichment analysis (ssGSEA) was performed using the GSVA package (v1.48.0) in R (v4.3.1). The input was the RNA-seq expression matrix of HAP-1 WT and USP21-KO cells (three biological replicates per group; SRA: PRJNA1026098), which was normalized and log_2_-transformed. Using the HALLMARK gene set collection from MSigDB as reference, enrichment scores of each pathway were calculated for every sample; for visualization, pathway scores were Z-score normalized across samples. A two-tailed unpaired Student’s *t*-test was applied to compare GSVA enrichment scores between the WT group and the USP21-KO group.

### 2.35 Statistical Analysis

All quantitative data are expressed as mean ± standard deviation (SD). Statistical analysis was performed with GraphPad Prism 10 (GraphPad Software, Boston, MA, USA). The unpaired two-tailed Student’s *t*-test was used for comparisons between two independent groups. One-way ANOVA followed by Tukey’s multiple-comparisons test was used for multi-group comparisons of single-time-point data. Two-way mixed-design ANOVA (treatment × time) was used for longitudinal datasets with repeated measurements from the same subjects (including tumor-volume and body-weight growth curves); Mauchly’s test was used to evaluate sphericity, and the Greenhouse–Geisser correction was applied when sphericity was violated. Tukey’s *post hoc* test was used for day-20 endpoint comparisons. Differences with a *p*-value less than 0.05 were regarded as statistically significant (*p < 0.05, **p < 0.01, ***p < 0.001, ****p < 0.0001).

## 3 Results

### 3.1 Virtual Screening and Structure-Based Optimization Identify USP21-in6

To identify potential USP21 inhibitors, we performed a structure-based hierarchical virtual screening of an FDA-approved drugs (n = 2662) (Fig. 1A). Initially, 100 compounds with favorable AutoDock Vina binding scores were prioritized. Further refinement using MM/PBSA binding free energy (<−100 kJ/mol) and ligand RMSD (<3.0 Å) yielded 10 lead candidates (Table S7, Fig. S1). Subsequent enzymatic validation with a ubiquitin-rhodamine-110 assay identified Tanshinone IIA Sulfonate as a USP21 inhibitor, with IC50 of 2.43 μM (Fig. 1E).

**Figure 1:**
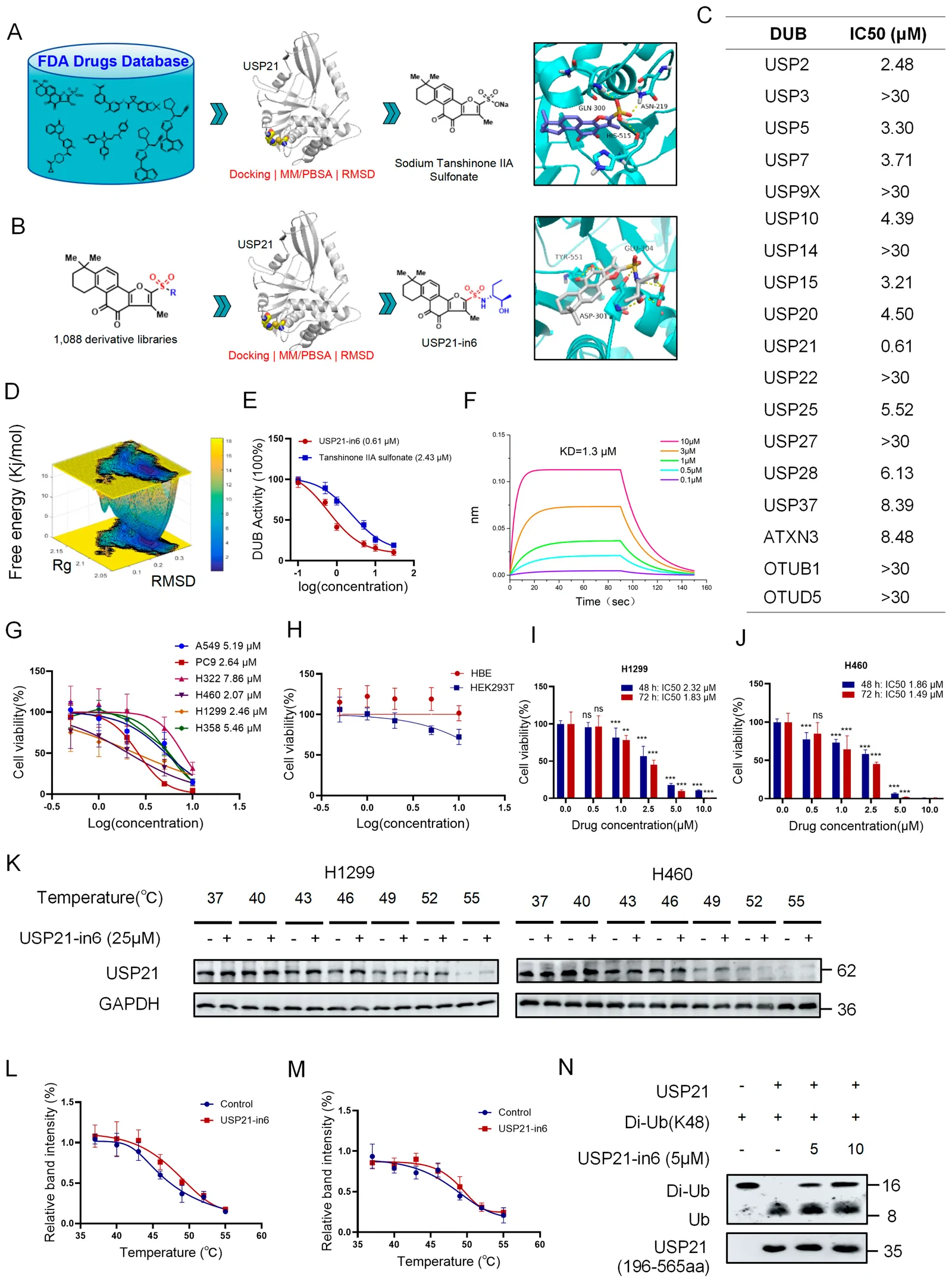
**Discovery and characterization of the USP21 inhibitor USP21-in6.** (**A**) Workflow for the virtual screening of an FDA-approved/natural product library, identifying sodium Tanshinone IIA Sulfonate as a lead USP21 inhibitor. (**B**) Structure-based optimization strategy. A library of 1088 derivatives was generated via systematic R-group substitution, leading to the identification of the optimized candidate USP21-in6. (**C**) Selectivity profile of USP21-in6. IC_50_ values against a panel of 18 DUBs. (**D**) Gibbs free energy landscape of the USP21-in6/USP21 complex. (**E**) Enzymatic inhibition of USP21 by USP21-in6. Dose-response curve from a ubiquitin-rhodamine 110 assay. (**F**) Binding kinetics of USP21-in6 to USP21. A representative biolayer interferometry (BLI) sensorgram shows real-time association and dissociation. (**G**) Cytotoxicity of USP21-in6 in NSCLC cell lines (A549, PC9, H1299, H460, H358, H322) after 24 h treatment (CCK-8 assay). Cell viability was normalized to the untreated control group. Since log(0) is mathematically undefined, the data point located at log(concentration) = −0.5 corresponds to the real-experimental 0 μM untreated control; a virtual nominal concentration was used exclusively for visualization and does not reflect actual drug concentration. (**H**) Cytotoxicity of USP21-in6 in normal cell lines (HBE, HEK293T) after 24 h treatment. (**I**,**J**) Time-dependent cytotoxicity of USP21-in6 in H1299 (**I**) and H460 (**J**) cells treated for 48 or 72 h. (**K**–**M**) Cellular thermal shift assay (CETSA) for target engagement. (**K**) Immunoblot of USP21 in H1299 and H460 cells treated with 25 μM USP21-in6 or DMSO across a temperature gradient (37–55°C). Quantification of USP21 thermal stabilization in H1299 (**L**) and H460 (**M**) cells. (**N**) USP21-in6 inhibits USP21-mediated cleavage of K48-linked di-ubiquitin. Protein molecular weights (unit: kDa) are labeled beside each target band. Data are mean ± SD (n = 3). Statistical significance was determined using a two-tailed unpaired Student’s *t*-test or one-way ANOVA. Comparisons were defined as follows: in (**H**), each USP21-in6 concentration group was compared with the matched 0 μM (DMSO vehicle) control within the same cell line; in (**I**,**J**), each concentration group was compared with the 0 μM control within the same treatment duration (48 h or 72 h), and the two time points at each concentration were additionally compared with each other; in (**L**,**M**), the USP21-in6-treated group was compared with the DMSO control group at each temperature. **p < 0.01; ***p < 0.001. ns, *p* ≥ 0.05. Abbreviation: FDA, Food and Drug Administration; USP, ubiquitin-specific protease; MM, molecular mechanics; PBSA, Poisson-Boltzmann surface area; RMSD, root-mean-square deviation; Gln, glutamine; Asn, asparagine; His, histidine; DUB, deubiquitinating enzyme; IC_50_, half-maximal inhibitory concentration; Rg, radius of gyration; KD, dissociation constant; ATXN, ataxin; OTUB, ovarian tumor-domain-containing ubiquitin-aldehyde-binding protein; OTUD, ovarian tumor-domain-containing deubiquitinase; GAPDH, glyceraldehyde-3-phosphate dehydrogenase; Di-Ub, di-ubiquitin; Ub, ubiquitin.

To improve potency, we structurally modified Tanshinone IIA Sulfonate. Structure-activity relationship studies revealed that removal of the sulfonate group (Tanshinone IIA) markedly reduced USP21 inhibitory activity (IC50 = 24.0 μM) (Fig. S2A,B). Retaining the sulfonate moiety, we conducted a structure-based R-group monosubstitution analysis using a library of 1088 aliphatic, aromatic, and heterocyclic substituents. This effort yielded the optimized analogue USP21-in6, which demonstrated enhanced enzymatic inhibition (IC50 = 0.61 μM; Fig. 1B,E). Kinetic binding assays revealed high affinity between USP21-in6 and USP21 (KD = 1.3 μM; Fig. 1F). Selectivity profiling across 18 DUBs revealed that USP21-in6 also inhibited USP2, USP5, USP7, and USP15 (IC50 values of 2.48, 3.30, 3.71, and 3.21 μM, respectively), while exhibiting no significant activity against USP3, USP9X, USP14, USP22, USP27, OTUB1 or OTUD5 at 30  μM. Of note, USP21-in6 also displayed measurable inhibitory activities against USP10, USP20, USP25, USP28, USP37 and ATXN3 in this *in vitro* DUB screening assay. These activities were not further characterized in this study (Fig. 1C and Fig. S3). To investigate the binding mode of USP21-in6, we conducted 50 ns molecular dynamics (MD) simulations. USP21-in6 achieved a stronger binding free energy (−157.53  kJ/mol) than Tanshinone IIA Sulfonate (−135.97 kJ/mol) (Table S8). The Gibbs free energy landscape further revealed a stable conformational distribution upon USP21-in6 binding (Fig. 1D). Per-residue energy decomposition analysis identified hotspot residues, suggesting that contributions from highly-conserved residues Val514 and His515 on the USP21 loop may contribute to inhibitor binding (Fig. S4). Although Val514 and His515 themselves are highly-conserved (ConSurf grades 8 and 9), distinct sequence features within the surrounding USP21 loop may underlie the inhibitor’s selectivity profile.

### 3.2 USP21-in6 Potently and Selectively Inhibits NSCLC Cell Proliferation

We next evaluated the pharmacological effects of USP21-in6 on six NSCLC cell lines (A549, H1299, H460, H358, PC9 and H322). CCK-8 assays after 24 h treatment demonstrated concentration-dependent inhibition of proliferation across all lines, with the most pronounced effects in H460 and H1299 cells (IC50 values of 2.07 and 2.46 μM, respectively; Fig. 1G). These potencies were substantially higher than those of the parent compound Tanshinone IIA Sulfonate. Prolonged exposure (48 and 72 h) further enhanced growth inhibition in a time-dependent manner (Fig. 1I–J). Importantly, USP21-in6 exhibited extremely low cytotoxicity among the normal cell lines tested, including HEK293T and HBE. (Fig. 1H).

Thermal shift assays in H460 and H1299 cells confirmed target engagement: Raw immunoblots indicated a tendency toward improved thermal stability of USP21 upon USP21-in6 treatment across the 37–55°C range, whereas CETSA curve fitting revealed only a minor thermal shift (Fig. 1K–M). Finally, immunoblotting with an anti-ubiquitin antibody revealed that USP21-in6 reduced USP21-mediated cleavage of di-ubiquitin to mono-ubiquitin, confirming functional inhibition of enzymatic activity in a cell-free biochemical assay (Fig. 1N).

### 3.3 Antitumor Effects of USP21-in6 Are Mediated Primarily via USP21

Dysregulation of DUBs is recognized as a hallmark of cancer and represents a promising therapeutic target. Building on this premise, we chose H460 and H1299, the two most drug-sensitive NSCLC cell lines identified in the preceding CCK-8 tests, for a systematic investigation into the antitumor activities of USP21-in6 across diverse malignant cellular phenotypes. Specifically, the compound exerted potent, concentration-dependent inhibition of colony formation, cell migration, and invasion, while concurrently inducing apoptosis (Figs. S5 and S6).

To confirm the specificity of these effects for USP21, we employed genetic knockdown of USP21 in the same cell lines (Fig. S7). Subsequent functional assays revealed a substantial attenuation of USP21-in6 activity in knockdown cells. Specifically, the inhibitory effects on clonogenicity (Fig. 2A–C), cell migration (Fig. 2D–F), and invasion (Fig. 2G–I) were attenuated. Similarly, the apoptosis-inducing capacity of USP21-in6 was also reduced upon USP21 knockdown. (Fig. 2J–L).

**Figure 2:**
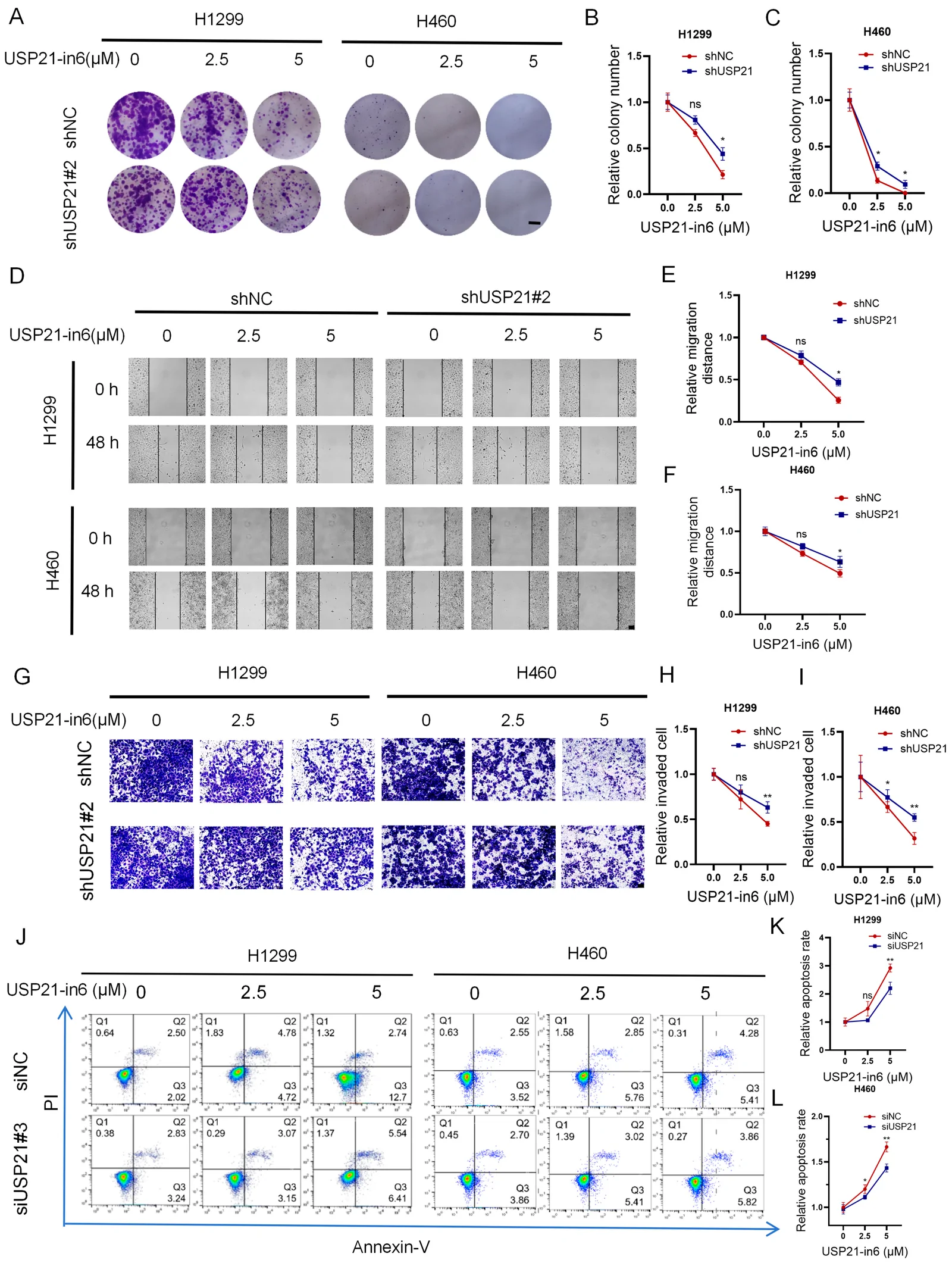
**USP21-in6 inhibits the proliferation, migration, and invasion of NSCLC cells and induces cell apoptosis by targeting USP21.** (**A**) Colony formation assay: H1299 and H460 cells were transfected with shNC or shUSP21. These cells were then treated with 0, 2.5, or 5 μM USP21-in6 and cultured for 14 days to allow colony formation. Scale bar = 3 mm. (**B**,**C**) Quantitative analysis of relative colony numbers. (**D**) Wound-healing assay: confluent H1299 and H460 cells (transfected with shNC or shUSP21) were scratched and treated with 0, 2.5, or 5 μM USP21-in6. Cell migration was imaged at 0 h, 24 h and 48 h. Scale bar = 100 μm. (**E**,**F**) Quantitative analysis of relative migration distance. (**G**) Transwell invasion assay: H1299 and H460 cells (transfected with shNC or shUSP21) were seeded in Matrigel-coated inserts and treated with 0, 2.5, or 5 μM USP21-in6 for 24 h. Scale bar = 100 μm. (**H**,**I**) Quantitative analysis of relative invaded cell numbers. (**J**) Apoptosis analysis (Annexin V/PI staining): H1299 and H460 cells (transfected with shNC or shUSP21) were treated with 0, 2.5, or 5 μM USP21-in6 for 24 h. The apoptosis rate was defined as the percentage of Annexin V-positive cells (early plus late apoptotic cells, Q2 + Q3). Q1: necrotic (Annexin V-/PI^+^) Q2: late apoptosis (Annexin V^+^/PI^+^) Q3: early apoptosis (Annexin V^+^/PI^−^) Q4: live (Annexin V^−^/PI^−^). Cells were then stained and analyzed by flow cytometry. (**K**,**L**) Quantitative analysis of the relative apoptosis rate. Data are mean ± SD (n = 3). For all relative measures, values were normalized to the matched negative-control (shNC or siNC) 0 μM group within the same cell line, which was set to 1. Statistical significance was determined using a two-tailed unpaired Student’s *t*-test. Comparisons included 2.5 μM and 5 μM USP21-in6 against 0 μM USP21-in6 within each shRNA or siRNA transfected group, as well as shNC versus shUSP21 under matched USP21-in6 concentrations. *p < 0.05; **p < 0.01; ns, *p* ≥ 0.05. Abbreviation: USP, ubiquitin-specific protease; shNC, short hairpin RNA negative control (non-targeting shRNA); shUSP21, short hairpin RNA targeting USP21; USP21-in6, USP21 inhibitor 6; siNC, small interfering RNA negative control; siUSP21, small interfering RNA targeting USP21; PI, propidium iodide.

### 3.4 USP21-in6 Suppresses Fatty Acid Synthesis by Downregulating Key Metabolic Enzymes

To elucidate the functional mechanisms of USP21 in NSCLC, we performed Gene Set Variation Analysis (GSVA) to evaluate signaling pathway activity upon USP21 knockout. Our GSVA results revealed a trend toward repressed fatty-acid-metabolism-associated pathway activity upon USP21 deletion (Fig. 3A), suggesting a potential role for USP21 in regulating lipid homeostasis. We therefore proceeded to examine whether pharmacological inhibition of USP21 influences fatty acid metabolism in NSCLC cells.

TEM revealed a marked reduction in lipid droplets in both H1299 and H460 cells following USP21-in6 treatment (Fig. 3B,C). Consistent with this, free fatty acid assays and Oil Red O staining indicated a dose-dependent decrease in neutral lipid accumulation and free fatty acid levels (Fig. 3D–F). BODIPY-based lipid quantification further confirmed a pronounced, concentration-dependent reduction in intracellular lipid content (Fig. 3G–J). Together, these results establish that USP21-in6 attenuates fatty acid levels in NSCLC cells through modulation of fatty acid metabolism.

Given that DNL accounts for approximately 90% of fatty acids in tumor cells, we focused on this pathway to dissect the underlying mechanism. qRT-PCR analysis showed that USP21-in6 did not alter the mRNA levels of USP21 or key lipogenic enzymes ACLY, ACC1, FASN, SCD1, and ACSL4 in H1299 and H460 cells after 24 h treatment (Fig. 3K,L). In contrast, Western blot analysis revealed a clear dose-dependent decrease in the protein expression of ACC1, FASN, SCD1, and ACLY, whereas the protein level of ACSL4 and USP21 remained unchanged (Fig. 3M). Collectively, these data indicate that USP21-in6 post-transcriptionally downregulates key enzymes of DNL without affecting USP21 expression, thereby linking USP21 inhibition to suppression of lipid anabolism in NSCLC.

**Figure 3:**
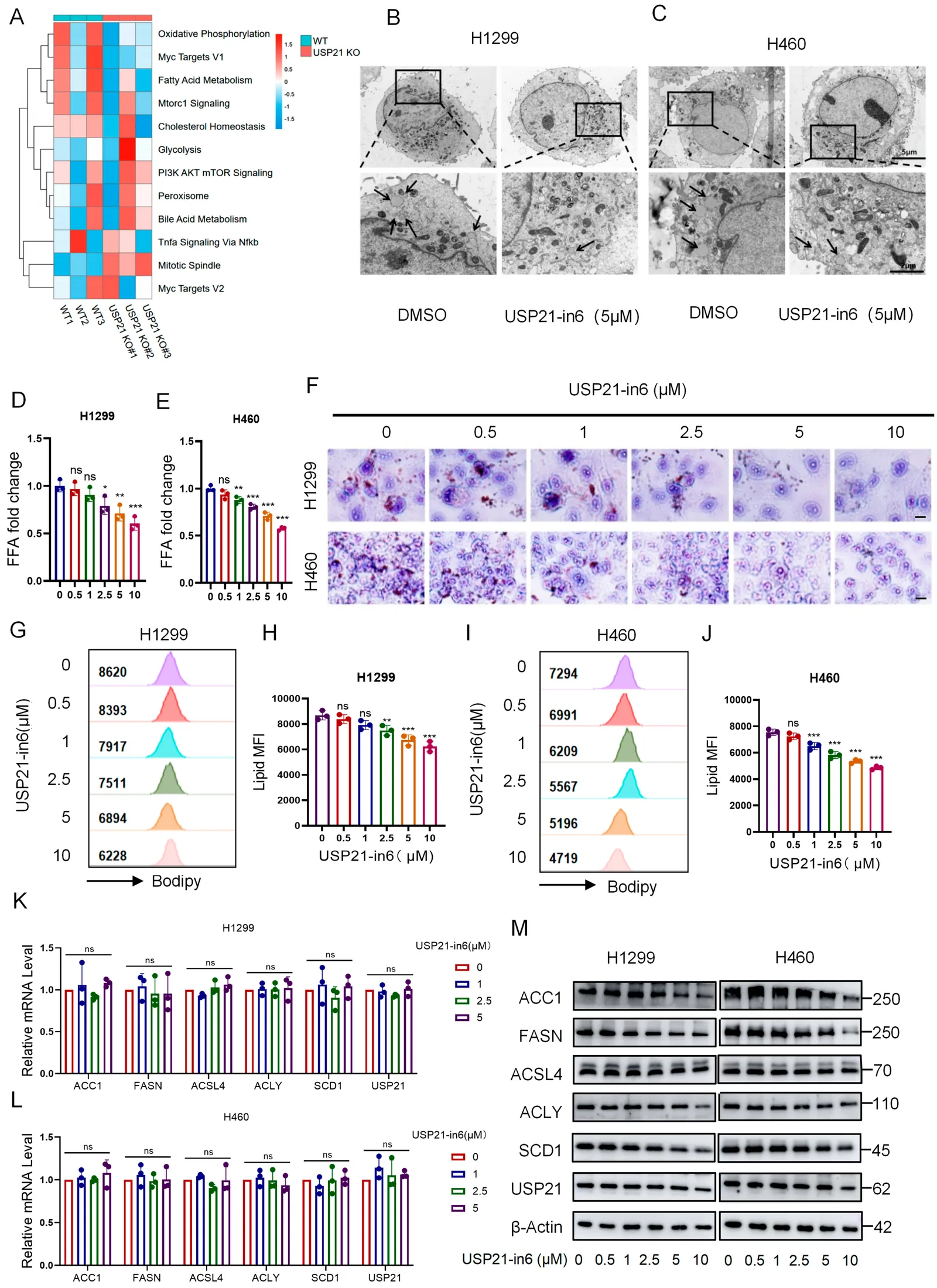
**USP21-in6 inhibits *de novo* fatty acid synthesis in NSCLC cells in a concentration-dependent manner.** (**A**) GSVA score heatmap of HALLMARK gene sets: compares wild-type (WT) and USP21 knockout (KO) cells, highlighting suppressed fatty acid metabolism pathways in USP21 KO cells. (**B**,**C**) TEM analysis: H1299 and H460 cells were treated with DMSO or 5 μM USP21-in6 for 24 h. Scale bars, 5 μm (upper panels); 2 μm (magnified lower panels). Arrows indicate lipid droplets. (**D**,**E**) Intracellular free fatty acid (FFA) levels: H1299 and H460 cells were treated with 0–10 μM USP21-in6 for 24 h. (**F**) Oil Red O staining: H1299 and H460 cells were treated with 0–10 μM USP21-in6 for 24 h. Representative images show reduced neutral lipid accumulation with increasing USP21-in6 concentration. Scale bar = 20 μm. (**G**,**I**) Flow cytometry analysis of lipid content (BODIPY): representative flow cytometry histograms of H1299 and H460 cells, treated with 0–10 μM USP21-in6 for 24 h. Shifts in fluorescence intensity reflect changes in intracellular lipid levels. (**H**,**J**) Quantitative analysis of relative lipid mean fluorescence intensity (MFI). (**K**,**L**) mRNA levels of *de novo* fatty acid synthesis enzymes: H1299 and H460 cells were treated with 0–5 μM USP21-in6 for 24 h. Relative mRNA levels of ACC1, FASN, ACSL4, ACLY, SCD1 and USP21. (**M**) Protein levels of *de novo* fatty acid synthesis enzymes: H1299 and H460 cells were treated with 0–10 μM USP21-in6 for 24 h. Immunoblotting detects USP21, ACC1, FASN, ACSL4, ACLY, and SCD1 protein levels. Protein molecular weights (unit: kDa) are labeled beside each target band. For all quantitative panels (**D**,**E**,**H**,**J**–**L**): data represent mean ± SD from n = 3 independent biological replicates. Statistical comparisons were performed versus the 0 μM USP21-in6 (DMSO) control group. *p < 0.05; **p < 0.01; ***p < 0.001, *ns p* ≥ 0.05. Abbreviation: WT, wild type; USP21, ubiquitin-specific peptidase 21; KO, knockout; PI3K, phosphoinositide 3-kinase; AKT, AKT serine/threonine kinase (protein kinase B); mTOR, mechanistic target of rapamycin; NF-κB, nuclear factor kappa B; DMSO, dimethyl sulfoxide; USP21-in6, USP21 inhibitor 6; FFA, free fatty acid; MFI, mean fluorescence intensity; ACC1, acetyl-CoA carboxylase 1; FASN, fatty acid synthase; ACSL4, acyl-CoA synthetase long-chain family member 4; ACLY, ATP citrate lyase; SCD1, stearoyl-CoA desaturase 1.

### 3.5 USP21 Deubiquitinates and Stabilizes SCD1

Next, Co-IP experiments revealed that USP21 specifically interacted with SCD1, but not with other key lipogenic enzymes including ACC1, FASN, and ACLY, in H1299 and H460 cells. Furthermore, the interaction between USP21 and SCD1 was verified in HEK293T cells (Fig. 4A,B). We next investigated the pathway responsible for SCD1 degradation using pathway-specific inhibitors. USP21 knockdown-induced SCD1 degradation was rescued by the proteasome inhibitor MG132, but not by the lysosomal inhibitor chloroquine, indicating that USP21 regulates SCD1 stability primarily through the ubiquitin-proteasome system (Fig. S8A–C). CHX chase assays showed that SCD1 protein half-life was prolonged upon USP21 overexpression in HEK293T cells (Fig. 4D,E), while USP21 knockdown shortened the SCD1 protein half-life in H1299 and H460 cells (Fig. S8D–F), confirming that USP21 stabilizes SCD1. Moreover, treatment with USP21-in6 significantly reduced SCD1 stability in a concentration-dependent manner, and this effect was reversed by MG132, consistent with USP21 knockdown phenotypes (Fig. S8G–Q). These results indicate that USP21-in6 disrupts SCD1 stability by inhibiting USP21-mediated stabilization.

We next examined whether USP21 acts as a deubiquitinase for SCD1. Co-expression of wild-type USP21, but not the catalytically inactive C221A mutant, markedly reduced SCD1 ubiquitination levels (Fig. 4C,I). Using ubiquitin mutants retaining only a single lysine residue, we observed that, upon USP21 transfection, K27- and K33-linked polyubiquitin signals on SCD1 decreased more markedly, whereas the other tested linkage types showed smaller reductions (Fig. 4J and Fig. S9). As this result derives from a single experiment, whether USP21 exhibits a preference for K27/K33 linkages remains to be further verified. Truncation mapping revealed that both the N-terminal and C-terminal domains of USP21 contribute to SCD1 binding (Fig. 4F,G). To identify key ubiquitination sites on SCD1, we performed site-directed mutagenesis and identified lysine 60 (K60) as a critical residue for SCD1 ubiquitination (Fig. 4H and Table S9).

Collectively, these data demonstrate that USP21 directly binds to and deubiquitinates SCD1, with an apparent preference for K27/K33 linkages that requires further confirmation, thereby stabilizing SCD1 protein levels. USP21-in6 counteracts this stabilization by inhibiting USP21 catalytic activity, thereby leading to accelerated SCD1 degradation via the proteasome.

**Figure 4:**
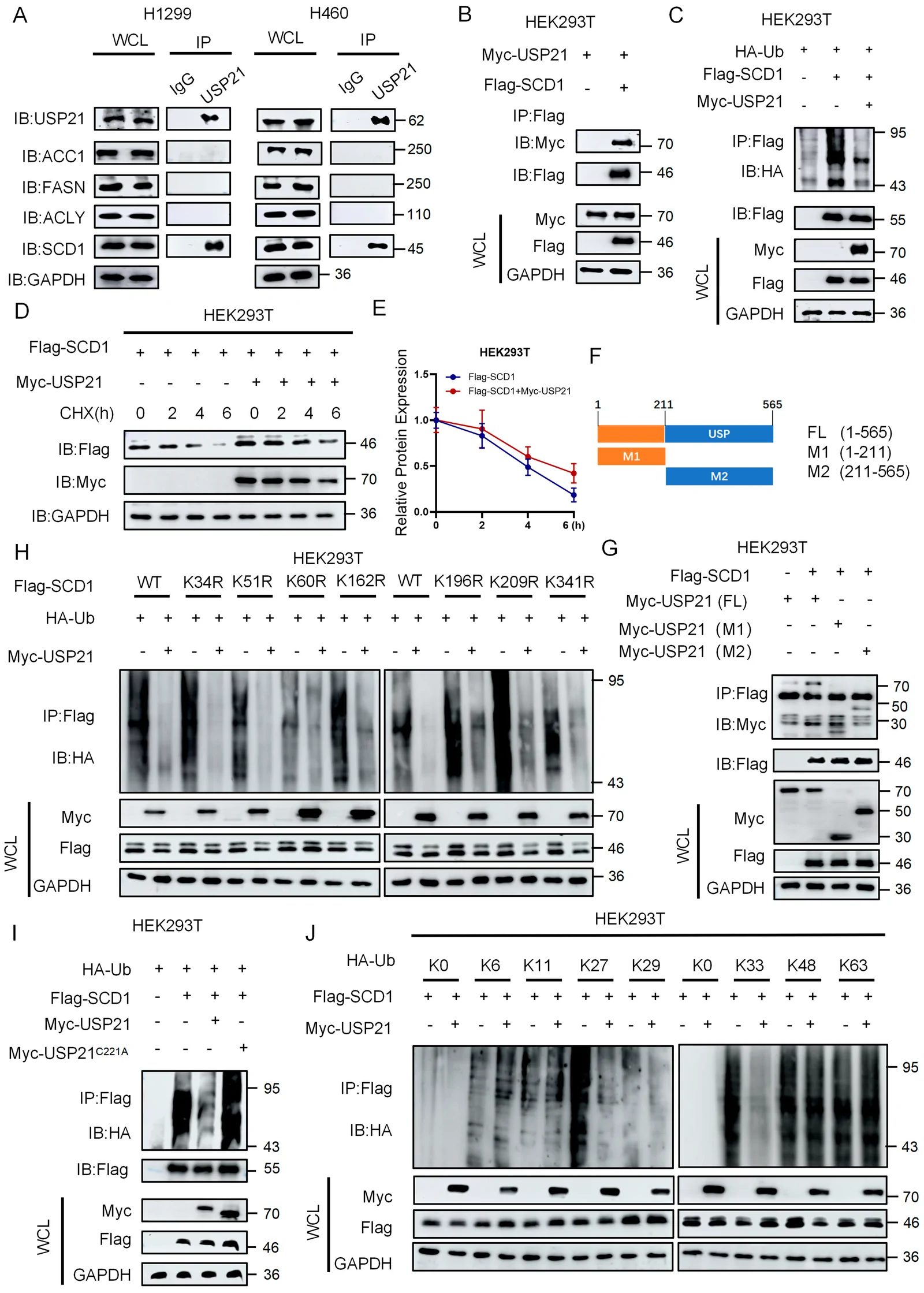
**USP21 interacts with SCD1 and regulates its stability via deubiquitination.** (**A**) Co-IP assay: whole-cell lysates (WCL) of H1299 and H460 cells were immunoprecipitated (IP) with IgG (control) or anti-USP21 antibody, followed by immunoblotting (IB) for SCD1 and USP21. (**B**) Co-IP assay in HEK293T cells: cells were transfected with Myc-USP21 and/or Flag-SCD1. IP with anti-Flag antibody followed by IB shows that USP21 binds to SCD1. (**C**) Deubiquitination assay: HEK293T cells were transfected with HA-Ub, Flag-SCD1, and Myc-USP21. IP with an anti-Flag antibody followed by IB (anti-HA) shows USP21 reduces SCD1 ubiquitination. (**D**,**E**) Protein stability assay: HEK293T cells were transfected with Flag-SCD1 with or without Myc-USP21, then treated with CHX for 0–6 h. (**D**) IB shows SCD1 protein half-life is extended by USP21. (**E**) Quantitative analysis of relative SCD1 protein expression. (**F**) Schematic of USP21 truncations. (**G**) Binding domain mapping: HEK293T cells were transfected with Flag-SCD1 and USP21 truncations (FL/M1/M2). IP with an anti-Flag antibody. (**H**) Ubiquitination site mapping: HEK293T cells were transfected with Flag-SCD1 (WT or lysine mutants), HA-Ub, and Myc-USP21. IP with an anti-Flag antibody. (**I**) HEK293T cells were transfected with HA-Ub, Flag-SCD1, and Myc-USP21 (or catalytically inactive Myc-USP21C221A). IP with an anti-Flag antibody followed by IB (anti-HA) shows USP21 reduces SCD1 ubiquitination (USP21C221A lacks this effect). (**J**) Linkage-specific deubiquitination assay: HEK293T cells were transfected with Flag-SCD1, Myc-USP21, and linkage-specific HA-Ub (K6/K11/K27/K29/K33/K48/K63). IP with anti-Flag antibody. Protein molecular weights (unit: kDa) are labeled beside each target band. Data are mean ± SD (n = 3). Statistical significance was determined using a two-tailed unpaired Student’s *t*-test or one-way ANOVA. Abbreviation: WCL, whole cell lysate; IP, immunoprecipitation; IgG, immunoglobulin G; USP21, ubiquitin-specific peptidase 21; IB, immunoblotting; ACC, acetyl-CoA carboxylase; FASN, fatty acid synthase; ACLY, ATP citrate lyase; SCD, stearoyl-CoA desaturase; GAPDH, glyceraldehyde-3-phosphate dehydrogenase; Myc, Myc epitope tag; Flag, FLAG epitope tag; HA, hemagglutinin epitope tag; HA-Ub, HA-tagged ubiquitin; CHX, cycloheximide; FL, full length.

### 3.6 USP21 Regulates Fatty Acid Metabolism in NSCLC Cells via Targeting SCD1

To investigate whether USP21 regulates DNL in NSCLC cells through SCD1, we conducted a series of experiments in H1299 and H460 cells with siRNA-mediated knockdown or overexpression of USP21 and SCD1, together with treatment with 10 μM SCD1-in (A939572, Bide-pharm, Cat. No. BD215757, Shanghai, China) for 24 h and supplementation with 150 μM oleic acid (OA, Aladdin, Cat. No. O108810, Shanghai, China), the direct product of SCD1, complexed with fatty-acid-free BSA for 48 h. Western blot analysis confirmed the efficiency of both knockdown and overexpression, thereby validating the reliability of our experiments (Fig. 5A,G).

Measurements of intracellular FFA levels showed that in both cell lines, overexpression of Flag-SCD1 and OA treatment increased lipid content, whereas knockdown of USP21 (siUSP21) decreased it. Notably, co-treatment with Flag-SCD1 or OA restored lipid levels compared with siUSP21 alone. In parallel, knockdown of SCD1 (siSCD1) or treatment with SCD1-in significantly reduced lipid content, while overexpression of Myc-USP21 increased it. Importantly, co-treatment of Myc-USP21 with siSCD1 or SCD1-in exhibited lower lipid content than Myc-USP21 overexpression alone (Fig. 5B,C,H,I). BODIPY staining yielded consistent results, showing the same regulatory patterns under the indicated treatments (Fig. 5D–F,J–L).

Collectively, these data demonstrate that USP21 modulates DNL in NSCLC cells by regulating SCD1.

**Figure 5:**
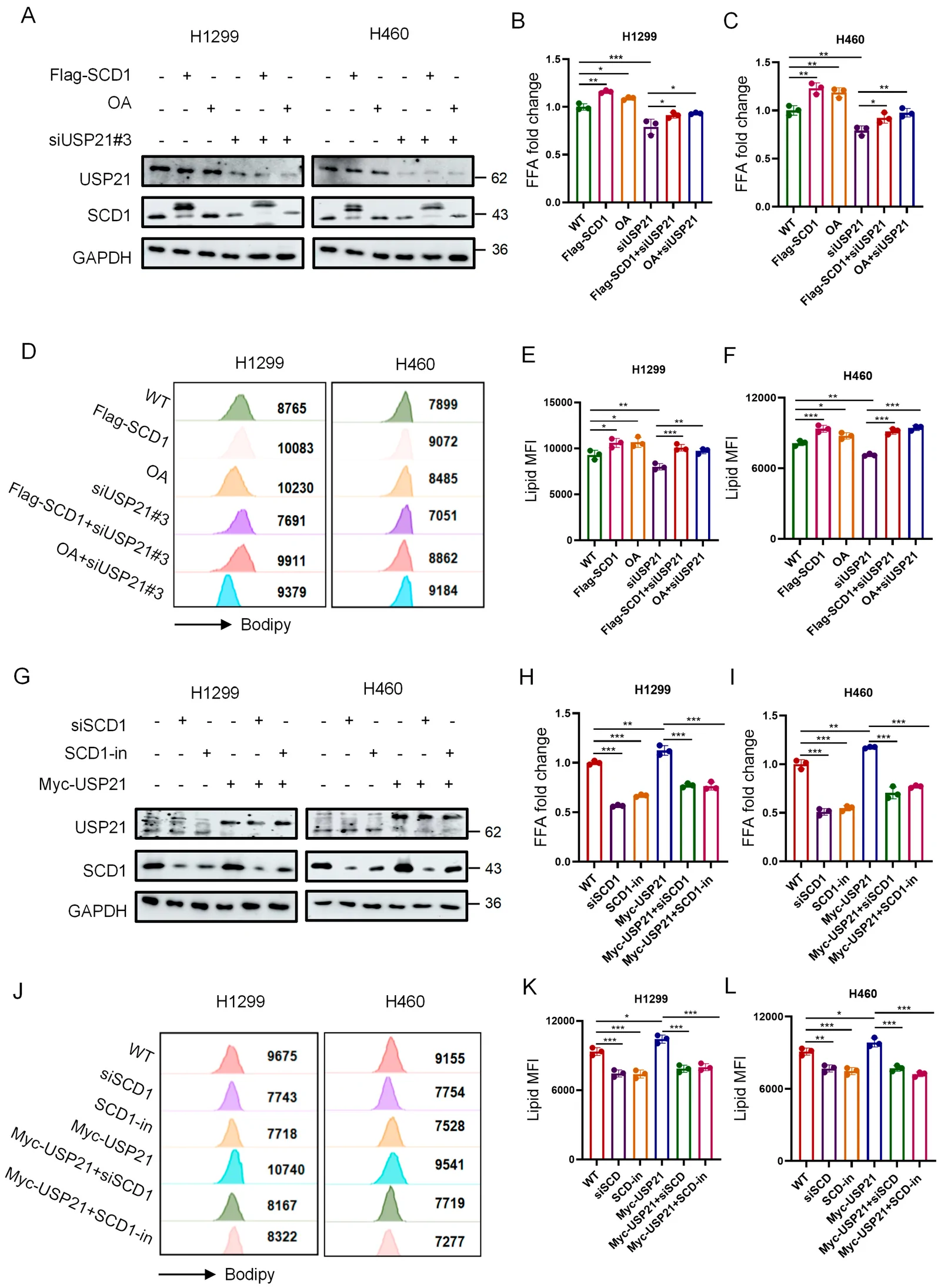
**USP21 regulates fatty acid metabolism in NSCLC cells via targeting SCD1.** (**A**) Immunoblot analysis: H1299 and H460 cells were assigned to groups (WT, Flag-SCD1 overexpression, OA treatment, siUSP21 knockdown, and combinations). Immunoblots show protein levels of USP21 and SCD1. (**B**,**C**) Relative free fatty acid (FFA) levels: H1299 (**B**) and H460 (**C**) cells were treated as in (**A**). FFA fold change is shown. (**D**) BODIPY staining for lipid levels: representative flow cytometry histograms of BODIPY-stained H1299 and H460 cells. Numbers indicate MFI of lipid signals. (**E**,**F**) Quantitative lipid MFI. (**G**) Immunoblot analysis: H1299 and H460 cells were assigned to groups (WT, siSCD1 knockdown, SCD1-in treatment, Myc-USP21 overexpression, and combinations). Immunoblots show protein levels of USP21 and SCD1. (**H**,**I**) Relative FFA levels: H1299 (**H**) and H460 (**I**) cells were treated as in (**G**). FFA fold change is shown. (**J**) BODIPY staining for lipid levels: representative flow cytometry histograms of BODIPY-stained H1299 and H460 cells. “WT” denotes untreated parental cells (no transfection, no treatment) and served as the baseline control for all groups. Flag-SCD1 and Myc-USP21 groups were transfected with the corresponding expression plasmids; siUSP21#3 and siSCD1 groups were transfected with gene-targeting siRNA. OA (150 μM) was delivered complexed with fatty-acid-free BSA for 48 h, and SCD1-in (10 μM) was dissolved in DMSO and applied for 24 h. Numbers indicate mean MFI of lipid signals. (**K**,**L**) Quantitative lipid MFI. Protein molecular weights (unit: kDa) are labeled beside each target band. For all quantitative panels (**B**,**C**,**E**,**F**,**H**,**I**,**K**,**L**): data are presented as mean ± SD, n = 3 independent biological replicates. Statistical analyses included both comparisons versus the WT baseline control and pre-defined pairwise comparisons between experimental groups. The displayed significance values correspond to these pairwise inter-group comparisons. Statistical significance was determined using a two-tailed unpaired Student’s *t*-test or one-way ANOVA. * *p* < 0.05; ** *p* < 0.01; ***p < 0.001. Abbreviation: USP21, ubiquitin-specific peptidase 21; SCD1, stearoyl-CoA desaturase 1; WT, wild type; OA, oleic acid; FFA, free fatty acid; MFI, mean fluorescence intensity; Bodipy, boron-dipyrromethene lipid dye; siUSP21, small interfering RNA targeting USP21; siSCD1, small interfering RNA targeting SCD1; SCD1-in, SCD1 inhibitor; Flag, FLAG epitope tag; Myc, Myc epitope tag; GAPDH, glyceraldehyde-3-phosphate dehydrogenase.

### 3.7 USP21-in6 Exhibits Potent Antitumor Activity and Favourable Safety In Vivo

To evaluate the *in vivo* therapeutic potential of USP21-in6, we utilized a syngeneic mouse model of NSCLC, specifically LLC cells in C57BL/6J mice. Given the established role of USP21 in stabilizing PD-L1 and evidence that SCD1 inhibition can activate immune cells, we designed combination regimens to probe both direct and immunomodulatory effects. Tumor-bearing mice were treated with USP21-in6 alone (10 or 20 mg/kg), an SCD1-in (10 mg/kg), an anti-PD-1 antibody (5 mg/kg), or their combinations (Fig. 6A).

USP21-in6 monotherapy significantly suppressed tumor growth in a dose-dependent manner (Fig. 6B,C). While SCD1-in or anti-PD-1 monotherapies showed moderate efficacy, their combination with USP21-in6 resulted in markedly superior tumor inhibition compared to any single agent. Safety assessment revealed no significant drug-induced necrosis in major organs (Fig. S10) and no abnormal elevation in serum AST or ALT levels (Fig. 6E,F), indicating that USP21-in6 possesses a favourable toxicity profile alongside its potent antitumor efficacy.

### 3.8 USP21-in6 Remodels the TME and Lipid Metabolism

Immunohistochemical analysis of excised tumors showed that the treatments, particularly combination therapies, markedly reduced the expression of the proliferation marker Ki-67 and of the target enzyme SCD1 (Fig. 6D). Furthermore, the combination of USP21-in6 with anti-PD-1 significantly enhanced the infiltration of CD4^+^ and CD8^+^ T cells into the tumor tissue (Fig. 6D).

Serum FFA assay showed FFA changes across groups (Fig. 6G). Lipidomic profiling revealed that treatment, especially combination regimens like USP21-in6 and SCD1-in, reshaped the lipid profiles in both serum and tumor tissues (Fig. 6H,I). Crucially, levels of the SCD1-catalyzed fatty acids C16:1 and C18:1 were specifically and significantly reduced in serum and tumors upon treatment, most notably in the combination groups (Fig. 6J–M). This confirms the on-target modulation of the USP21-SCD1-lipid synthesis axis *in vivo*.

**Figure 6:**
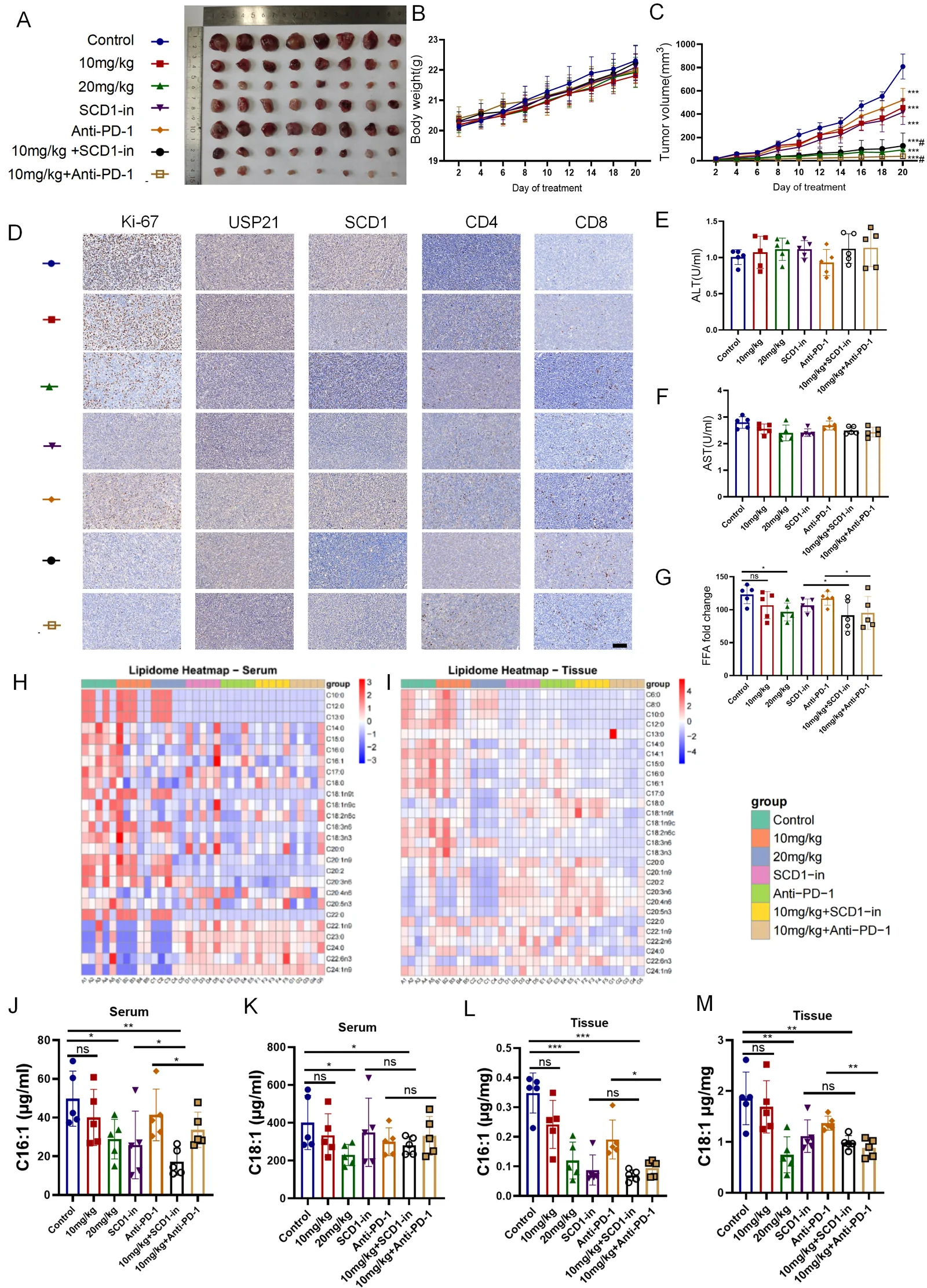
**USP21-in6 inhibits NSCLC in LLC tumor-bearing mice.** (**A**) *In vivo* tumor syngeneic assay: representative images of tumors from mice treated with vehicle (Control), 10 mg/kg USP21-in6, 20 mg/kg USP21-in6, SCD1-in, anti-PD-1, 10 mg/kg USP21-in6+SCD1-in, or 10 mg/kg USP21-in6+anti-PD-1. (n = 8 mice per group). (**B**) Body weight monitoring: mice body weight (g) was measured over 20 days of treatment. Data are presented as mean ± SD (n = 8 mice per group). No significant weight loss was observed across groups. (**C**) Tumor volume kinetics: tumor volume (mm^3^) was measured every 2 days during treatment. Combination groups show enhanced tumor growth inhibition. (n = 8 mice per group) tumor growth curves (**B**,**C**) were analyzed by two-way mixed-design ANOVA with Tukey’s multiple-comparisons test; single-time-point data were analyzed by one-way ANOVA or unpaired two-tailed Student’s *t*-test. In (**C**), ***p < 0.001 versus control; ^#^*p* < 0.001, combination group versus corresponding single-agent group. (**D**) Immunohistochemical (IHC) staining: tumor sections stained for Ki-67 (proliferation), USP21, SCD1, CD4^+^ T cells, and CD8^+^ T cells (representative images; scale bar = 100 μm). (**E**,**F**) Serum biochemistry: serum levels of ALT and AST (U/L) in treated mice, indicating no overt hepatic toxicity. (n = 5 mice per group). (**G**) Relative free fatty acid (FFA) levels: serum FFA fold change (vs. control group) in treated mice. (n = 5 mice per group). (**H**,**I**) lipidome heatmaps: serum (**H**) and tumor tissue (**I**) lipid profiles (normalized intensity; red = upregulated, blue = downregulated) across treatment groups. (**J**–**M**) Quantitative lipid analysis: serum levels of C16:1 (**J**) and C18:1 (**K**), and tumor tissue levels of C16:1 (**L**) and C18:1 (**M**) (μg/mL or μg/mg tissue). (n = 5 mice per group). For all quantitative panels: data are presented as mean ± SD. Statistical analyses included both comparisons versus the control group and pre-defined pairwise comparisons between different treatment groups. Tumor growth curves (**B**,**C**) were analyzed by two-way mixed-design ANOVA with Tukey’s multiple-comparisons test; single-time-point data were analyzed by one-way ANOVA or unpaired two-tailed Student’s *t*-test. *p < 0.05; **p < 0.01; ***p < 0.001, ns *p* ≥ 0.05. Abbreviation: USP21, ubiquitin-specific peptidase 21; PD-1, programmed death-1; SCD1, stearoyl-CoA desaturase 1; SCD1-in, SCD1 inhibitor; Ki-67, marker of proliferation Ki-67; CD4, cluster of differentiation 4; CD8, cluster of differentiation 8; ALT, alanine aminotransferase; AST, aspartate aminotransferase; FFA, free fatty acid.

### 3.9 USP21-in6 Reinvigorates Antitumor Immunity in an Immunologically Cold Model

To dissect the immunomodulatory mechanisms, we performed detailed flow cytometric analysis of tumor-infiltrating lymphocytes (TILs). Compared with the control group, treatment with USP21-in6 or SCD1-in significantly increased the infiltration of both CD4^+^ and CD8^+^ T cells, and the most prominent elevation was observed in the USP21-in6 plus anti-PD-1 combination group, with CD4^+^ and CD8^+^ T-cell percentages reaching 30.8% and 22.8%, respectively (Fig. 7A–E).

To assess functional status, we found that treatment with USP21-in6 and/or SCD1-in, increased the proportions of Granzyme B^+^ and IFN-γ^+^ CD8^+^ T cells within tumors (Fig. 7A–E). Notably, the combination of USP21-in6 with anti-PD-1 increased the frequencies of both Granzyme B^+^ and IFN-γ^+^ CD8^+^ T cells, indicating a restoration of cytotoxic function.

### 3.10 USP21-in6 Suppresses Tregs via Targeting the USP21-Foxp3 Axis

We next assessed the impact of USP21-in6 on immunosuppressive Tregs (CD4^+^CD25^+^Foxp3^+^) within the TME. Flow cytometric analysis revealed that USP21-in6 treatment, alone or in combination, significantly decreased intratumoral Treg frequency. In contrast, monotherapy with the SCD-in showed no significant effect, whereas anti-PD-1 treatment was associated with an increased Treg proportion (Fig. 7F–H). Consistent with this reduction in Treg abundance, Foxp3 protein levels in sorted Tregs exhibited a dose-dependent decrease following USP21-in6 treatment. Notably, although anti-PD-1 monotherapy robustly increased the frequencies of intratumoral CD4^+^ and CD8^+^ T cells as well as Granzyme B- and IFN-γ-producing cytotoxic CD8^+^ T cells, it also significantly elevated the proportion of immunosuppressive Foxp3^+^ Tregs within the tumor microenvironment. This concurrent expansion of Treg populations may counteract the anti-tumor potency of activated effector T cells, providing a potential explanation for the modest tumor-suppressive effect of anti-PD-1 monotherapy in our model, consistent with the known immunotherapy resistance of LLC cells.

This result carries important mechanistic implications. Previous studies have established that USP21 functions as a deubiquitinase for Foxp3, stabilizing its protein level and thereby maintaining Treg suppressive function. Our findings suggest that USP21-in6, by inhibiting USP21, disrupts this stabilization, leading to decreased Foxp3 expression, impaired Treg function, and reduced Treg accumulation within the TME. Thus, the mechanism of USP21-in6 extends beyond metabolic regulation via SCD1 to encompass direct immunomodulation through the Foxp3/Treg axis. In peripheral immune organs (spleen and lymph nodes), only minor fluctuations in Treg proportions were observed across treatment groups (Fig. 7I,J), indicating that the immunomodulatory effect of USP21-in6 is localized to the tumor site and does not induce systemic immune alterations, a finding that is consistent with its favorable safety profile.

**Figure 7:**
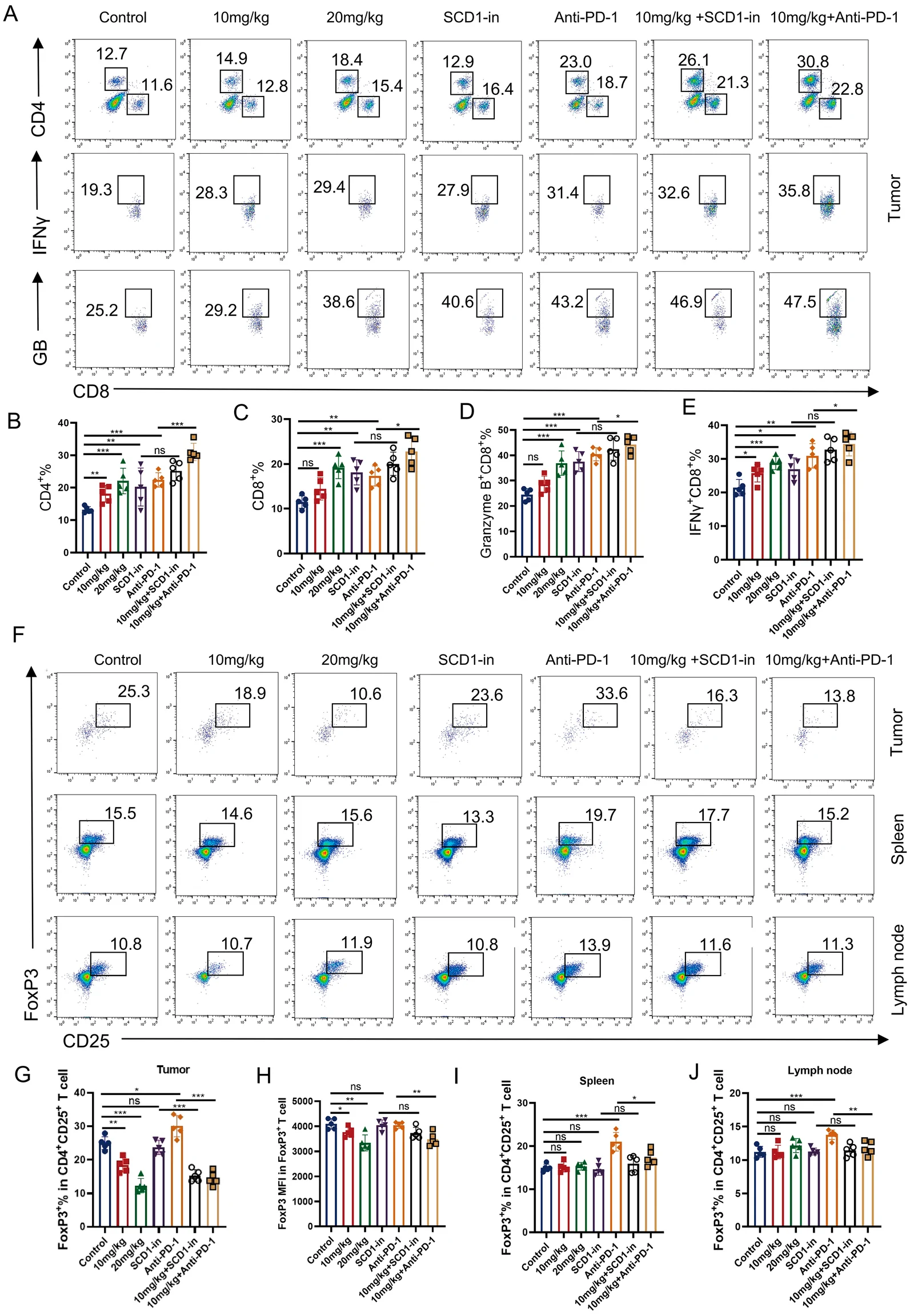
**USP21-in6 enhances anti-tumor immunity in LLC tumor-bearing mice.** (**A**) Flow cytometry analysis of tumor-infiltrating immune cells: representative dot plots of CD4^+^ T cells, IFNγ^+^ cells, and granzyme B (GB)^+^ cells (gated on CD8^+^ T cells) in tumors from mice treated with control, 10 mg/kg USP21-in6, 20 mg/kg USP21-in6, SCD1-in, Anti-PD-1, 10 mg/kg USP21-in6+SCD1-in, or 10 mg/kg USP21-in6+Anti-PD-1 (numbers = percentage of positive cells). (**B**–**E**) quantitative analysis of tumor-infiltrating immune cells: percentages of CD4^+^ T cells (**B**), CD8^+^ T cells (**C**), Granzyme B^+^ CD8^+^ T cells (**D**), and IFNγ^+^ CD8^+^ T cells (**E**) in tumors. (**F**) Flow cytometry analysis of Treg cells: representative dot plots of Foxp3^+^ CD25^+^ CD4^+^ T cells in tumor, spleen, and lymph node tissues from treated mice (numbers = percentage of treg cells). (**G**–**J**) Quantitative analysis of Treg cells: percentages of Foxp3^+^ CD25^+^ CD4^+^ T cells in tumor (**G**), spleen (**I**), and lymph node (**J**) tissues. Foxp3 MFI in Foxp3^+^ T cells in tumor (**H**). For all quantitative panels (**B**–**E**,**G**–**J**): data are presented as mean ± SD, n = 5 mice per group. Statistical analyses included both comparisons versus the control group and pre-defined pairwise comparisons between different treatment groups. Statistical significance was determined using a two-tailed unpaired Student’s *t*-test or one-way ANOVA. *p < 0.05; **p < 0.01; ***p < 0.001, ns *p* ≥ 0.05. Abbreviation: PD-1, programmed death-1; SCD1-in, stearoyl-CoA desaturase 1 inhibitor; CD4, cluster of differentiation 4; CD8, cluster of differentiation 8; IFNγ, interferon gamma; GB, granzyme B; Foxp3, forkhead box P3; CD25, cluster of differentiation 25 (interleukin-2 receptor alpha); MFI, mean fluorescence intensity; SCD1, stearoyl-CoA desaturase 1.

### 3.11 USP21 Expression Predicts Immunotherapy Response and Is Linked to SCD1 Levels in NSCLC

In summary, our findings establish that USP21 post-translationally regulates SCD1 protein expression, a relationship that was further validated *in vivo*. To examine the USP21-SCD1 axis in a clinical setting, we analyzed tissue microarrays comprising 80 paired NSCLC and adjacent non-tumor samples. Immunohistochemical staining revealed that both USP21 and SCD1 protein levels were significantly elevated in tumor tissues compared with their non-tumor counterparts, and their expression levels were positively correlated with each other (Fig. 8A–D).

To explore the potential clinical relevance of this axis in immunotherapy, we collected pre-treatment lung biopsy specimens from six NSCLC patients who received PD-1 inhibitor therapy (Fig. 8E–I, Table S10). Tumor response was assessed according to the Response Evaluation Criteria in Solid Tumors version 1.1 (RECIST 1.1). Contrast-enhanced chest and abdominal CT scans were performed for response evaluation after two cycles of anti-PD-1 monotherapy, followed by a 6-month follow-up. Patients were classified as responders (complete response or partial response) or non-responders (stable disease or progressive disease). All patients received anti-PD-1 monotherapy, including pembrolizumab, nivolumab, sintilimab, camrelizumab, tislelizumab, and penpulimab. All agents were administered at standard recommended doses in accordance with the clinical guidelines for NSCLC. Based on clinical response evaluation, three patients were classified as “responders” and three as “non-responders”. Notably, this small cohort (n = 6) limits definitive clinical validation, and our findings represent preliminary exploratory correlations. We observed that responsive patients exhibited robust intratumoral CD8^+^ T-cell infiltration, whereas non-responsive tumors showed concurrent upregulation of USP21 and SCD1.

**Figure 8:**
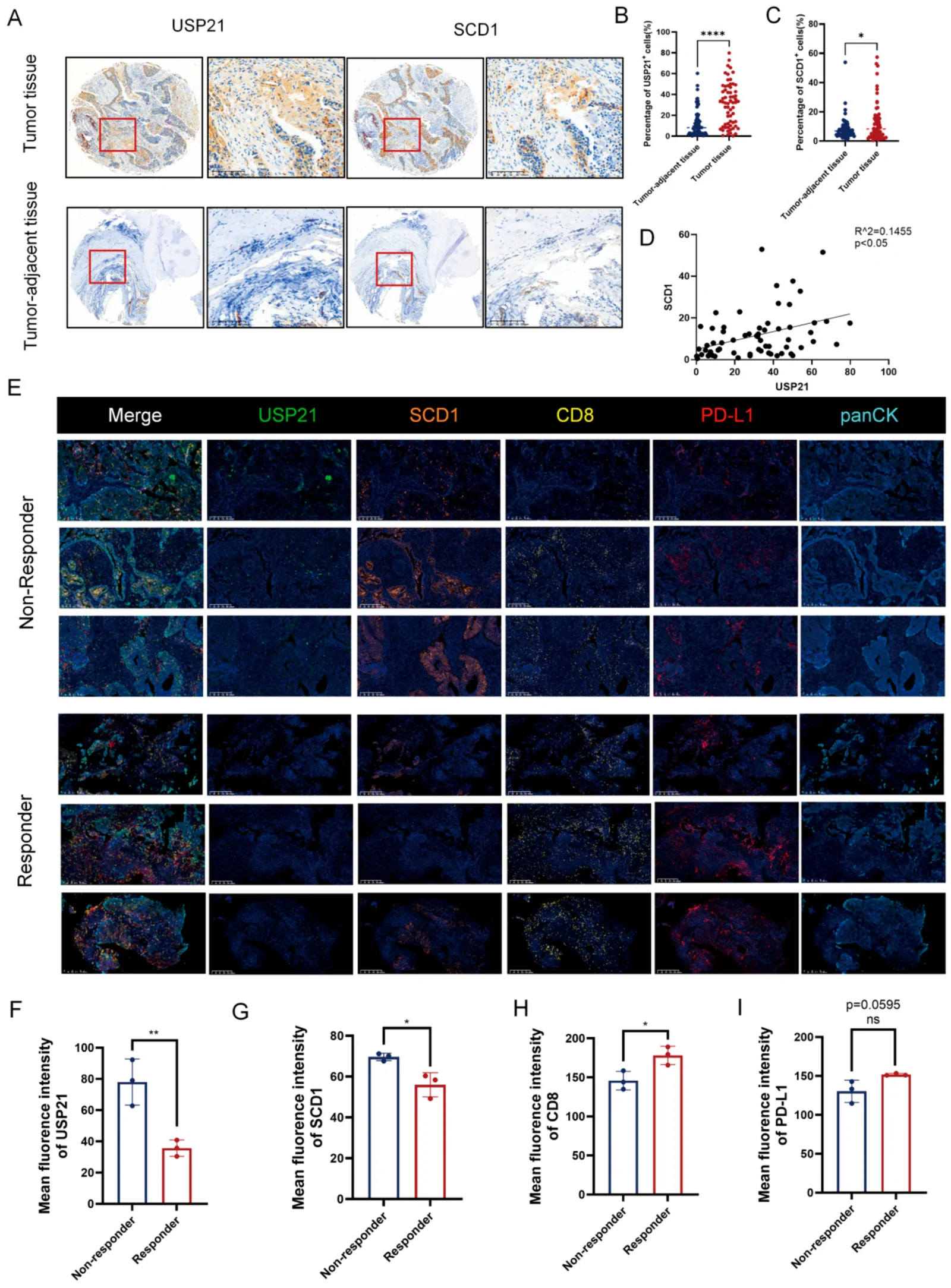
**Expression of USP21 and SCD1 in human NSCLC tissues.** (**A**) Immunohistochemical (IHC) staining: USP21 and SCD1 expression in tumor tissue vs. tumor-adjacent normal tissue from NSCLC patients; red boxes indicate magnified regions (representative images). Scale bar = 100 μm (**B**,**C**) percentage of USP21-positive cells and SCD1-positive cells in tumor vs. tumor-adjacent tissue. (**D**) Correlation scatter plot showing positive correlation between USP21 and SCD1 expression in tumor tissues. Simple linear regression, n = 80; Pearson’s r = 0.381, R^2^ = 0.1455, *p* < 0.05. (**E**) mIF staining: co-expression of USP21, SCD1, CD8 and PD-L1 (with DAPI) in tumor tissues from therapy non-responders vs. responders (representative images). Scale bar = 200 μm. (**F**–**I**) Quantitative fluorescence intensity: mean intensity of USP21, SCD1, CD8, and PD-L1 in non-responder vs. responder tissues. Data are presented as mean ± SD. Sample sizes and comparison groups were as follows: (**B**,**C**) paired tumor versus matched tumor-adjacent tissues from 80 NSCLC patients (Wilcoxon matched-pairs signed-rank test, n = 80 pairs); (**D**) correlation analysis of 80 tumor samples (simple linear regression, n = 80); (**F**–**I**) responder versus non-responder pretreatment tumor biopsies (two-tailed unpaired Student’s *t*-test, n = 3 patients per group). For comparisons between two independent groups (responder vs. non-responder), two-tailed unpaired Student’s *t*-test was applied. For paired tumor and adjacent non-tumor tissue samples, Wilcoxon matched-pairs signed-rank test was used because the data did not conform to normal distribution. Simple linear regression was performed to evaluate the correlation between USP21 and SCD1 expression. *p < 0.05; **p < 0.01; ****p < 0.0001, *ns p* ≥ 0.05. Abbreviation: USP21, ubiquitin-specific peptidase 21; SCD1, stearoyl-CoA desaturase 1; CD8, cluster of differentiation 8; PD-L1, programmed death-ligand 1; panCK, pan-cytokeratin.

## 4 Discussion

The advent of targeted therapies and ICIs has significantly reshaped the clinical treatment landscape for NSCLC. However, primary and acquired resistance-driven by intrinsic oncogenic signaling plasticity and an immunosuppressive TME-remains a core challenge in current clinical practice [54,55]. This clinical predicament underscores the urgency of developing novel therapeutic strategies, with molecular targets capable of simultaneously disrupting tumor cell survival pathways and reprogramming the immunosuppressive microenvironment emerging as a central research focus. DNL, a core metabolic hallmark of tumors, features key enzymes that serve as pivotal nodes linking lipid metabolism and tumor immunity. The UPS’s regulation of metabolic enzyme stability offers a novel avenue for identifying upstream critical molecular nodes in this field.

By adopting a directed optimisation strategy starting from clinically safe drugs to develop USP21-in6, this study effectively reduces the risks and costs associated with new drug development, providing an exemplary model for identifying novel therapeutic targets for cancer treatment from existing drug libraries. Tanshinone IIA sulfonate, a clinically established cardiovascular therapeutic with a well-defined safety profile, served as the structural basis. Through structural modification and structure-activity relationship analysis, we obtained the optimized derivative USP21-in6. *In-vitro* biochemical experiments show that the inhibitor exhibits sub-micromolar inhibitory activity against USP21. Admittedly, it also suppresses multiple deubiquitinases, including USP2, USP5, USP7 and USP15. The IC_50_ values of these off-target enzymes differ from those of USP21 by only 4- to 6-fold than that for USP 21, indicates a modest selectivity window, with the most prominent off-target inhibition observed for USP2. Although full-length USP2 and USP21 share around 50% overall sequence identity, the high-conservation of their catalytic domains leads to cross-inhibition. As such, potential phenotypic interference resulting from USP2 suppression cannot be fully ruled out. MD simulations confirmed that USP21-in6 forms stable complexes with USP21, with its selectivity potentially arising from specific interactions with loop-specific residues that differ among USP family members. Cell-based functional assays revealed that USP21-in6 could effectively suppress the proliferation, migration and invasion of NSCLC cell lines. Within the tested concentration range, it exhibited low cytotoxicity against the two types of normal cells examined in this study. Thermal shift assays and ubiquitin-cleavage experiments further verified its intracellular target-binding capacity and functional inhibitory activity, laying a solid foundation for subsequent *in-vivo* experiments and clinical translation.

This study first identifies USP21 as a natural deubiquitinase of SCD1. Functional experiments show that USP21-in6 treatment markedly reduces the protein levels of multiple lipogenic enzymes, including ACC1, FASN, and ACLY. However, Co-IP results verify that USP21 directly interacts only with SCD1, but not with ACC1, FASN, or ACLY. USP21-in6-induced SCD1 depletion reduces intracellular C16:1 and C18:1 levels and disrupts global lipid homeostasis. Notably, our qPCR results (Fig. 3K,L) demonstrate unaltered mRNA levels of ACC1, FASN, and ACLY after USP21-in6 treatment, indicating that the reduction of these lipogenic enzymes occurs independently of transcriptional repression. Instead, this disturbed lipid homeostasis may trigger post-transcriptional or post-translational feedback regulation to decrease ACC1, FASN and ACLY protein abundance, consistent with previous reports describing feedback effects upon SCD1 depletion [56,57,58]. Collectively, we speculate that the downregulation of lipogenic enzymes following USP21-in6 treatment is primarily a secondary feedback consequence induced by SCD1 depletion, rather than a direct effect caused by the broad off-target inhibition of USP21-in6. This finding substantially expands the functional spectrum of USP21, extending its previously reported regulatory substrates (PD-L1 and YY1) to the core regulatory pathways of tumor lipid metabolism [41,42]. For instance, overexpression of FASN, a key enzyme in DNL, correlates negatively with immune cell infiltration across 35 tumor types. Moreover, the fatty acid synthesis it mediates is essential for Treg functional maturation, FASN knockout in Treg cells specifically enhances anti-tumor immunity [8]. ACLY exerts bidirectional regulation: its inhibition promotes anti-tumor immunity by activating the cGAS-STING pathway, yet simultaneously upregulates PD-L1 expression and induces elevated T-cell exhaustion markers LAG3 and TIGIT, ultimately impairing immunotherapy response [9,10]. In contrast, ACSL4 exerts distinct pro-immune properties in tumors such as colorectal and bladder cancers. Knockdown of ACSL4 activates the RIG-I-MAVS-type I interferon pathway, enhancing CD8^+^ T-cell infiltration and activation. Consequently, tumors lacking ACSL4 demonstrate markedly improved response to PD-L1 blockade therapy [11,12]. As the key rate-limiting enzyme in DNL, SCD1 catalyzes the production of monounsaturated fatty acids, which are essential for maintaining membrane fluidity, signal transduction, and energy storage in tumor cells [59,60,61,62]. Serving as a pivotal link between lipid metabolism and tumor cell survival, its functional abnormalities play an irreplaceable role in the progression of malignant tumors such as NSCLC.

This study further validated the clinical relevance of the USP21-SCD1 axis. Analysis of tissue microarrays comprising 80 paired NSCLC and adjacent non-tumor samples revealed significantly elevated and positively correlated expression of both USP21 and SCD1 proteins. Analysis of six NSCLC patient samples treated with PD-1 inhibitors revealed that non-responders exhibited concurrent of USP21 and SCD1 in tumor tissues, accompanied by reduced CD8^+^ T-cell infiltration. This suggests the USP21-SCD1 axis not only serves as a key driver of NSCLC progression but also holds potential as a biomarker for predicting immunotherapy response, offering guidance for stratified clinical treatment. This finding aligns closely with clinical characteristics observed in other DNL key enzymes, such as the ACLY-H3K27ac-PD-L1 axis, which effectively predicts poor immunotherapy outcomes [9]. It further substantiates the close association between the expression levels of DNL key enzymes and their upstream regulatory molecules and tumor immunotherapy efficacy, while highlighting the potential of the USP21-SCD1 axis as a clinically valuable predictive and prognostic marker.

Previous studies have shown that USP21 inhibition destabilizes Foxp3, impairs the function of regulatory T-cells, blocks their recruitment to the TME, and ultimately relieves tumor-mediated immune suppression [46,47]. Accordingly, the dual mechanism of USP21-in6 may account for its capacity to boost the therapeutic efficacy of anti-PD-1 treatment. By contrast, other key enzymes involved in DNL merely regulate specific lipid-metabolic pathways in a unilateral manner and cannot act directly on immune cells. For instance, although FASN is associated with Treg cell function, it modulates cellular activity only indirectly via fatty-acid synthesis [63]. These distinctions highlight that targeting USP21 holds greater translational potential than targeting individual rate-limiting enzymes of DNL.

Thus, USP21-in6 achieves dual regulation of tumors: first, by degrading SCD1 to sever the lipid metabolic supply to tumor cells, undermining their core foundation for survival and proliferation, and second, by attenuating Foxp3-mediated immune suppression to enhance the anti-tumor killing capacity of CD8^+^ T cells. This approach concurrently addresses two major barriers to NSCLC immunotherapy resistance: metabolic support and immune evasion. This may explain the improved efficacy of anti-PD-1 therapy observed upon USP21-in6 co-administration *in vivo*. Beyond its mechanistic actions, *in vivo* studies further support the clinical potential of USP21-in6. The compound exhibits dose-dependent antitumor efficacy in syngeneic NSCLC mouse models, alongside a favorable safety profile. No significant drug-induced organ damage was observed across major organs, nor were abnormal elevations in serum transaminase levels detected. This provides robust safety evidence for subsequent preclinical studies and clinical translation.

### Limitations

Due to experimental conditions and study design, the present work has several inherent limitations. First, cell models with gradient USP21 expression were not established for quantitative correlation analysis, and the intrinsic relationship between endogenous USP21 abundance and cellular sensitivity to USP21-in6 remains undetermined. Second, cytotoxicity tests were conducted merely on two immortalized normal cell lines. Long-term *in vivo* toxicological observation and validation using primary normal cells are currently lacking, which limits the current safety assessment. Third, USP21-in6 exerts moderate off-target inhibition of the homologous deubiquitinase USP2, and its target selectivity requires further optimization. Only preliminary *in vivo* efficacy studies have been performed to date, and systematic pharmacokinetic profiles, including absorption, distribution, metabolism, and excretion (ADME) properties, have not yet been characterized, leaving the *in vivo* behavior of this compound largely undefined. Fourth, we did not assess the nuclear translocation of SREBP1c; therefore, the complete feedback regulatory mechanisms governing downstream lipid metabolism have not been fully delineated. Lastly, the evaluation of therapeutic responses to PD-1-based immunotherapy was limited to six clinical samples, which constrains the statistical power and generalizability of our clinical observations. The small sample size and the absence of an independent validation cohort make it difficult to verify the reliability of the USP21-SCD1 axis as a prognostic biomarker for immunotherapy. In future studies, we plan to further optimize the compound structure to reduce off-target effects and improve target selectivity. Additional mechanistic investigations will be undertaken to elucidate the SREBP1c-mediated feedback regulatory network governing lipid metabolism. In-depth preclinical pharmacokinetic evaluation, long-term toxicological profiling, and safety validation using primary normal cells are also planned. Larger-scale, multicenter clinical cohorts will be established to further corroborate the predictive value of the USP21-SCD1 axis in immunotherapy response. Moreover, we aim to explore the regulatory relationships between USP21 and other key lipogenic enzymes, as well as to refine combination therapeutic strategies, with the goal of providing preliminary experimental evidence and a mechanistic framework to support immunometabolic targeted therapy in NSCLC.

## 5 Conclusion

In summary, this study demonstrates that USP21 functions as a deubiquitinase of SCD1 and regulates both tumor lipid metabolism and tumor immunity in NSCLC. USP21 maintains tumor lipogenesis by stabilizing SCD1 protein. Meanwhile, it modulates Foxp3-dependent function and regulates immune suppression within the TME, thereby partly affecting malignant progression and immunotherapy sensitivity of NSCLC. The small-molecule USP21-targeted inhibitor USP21-in6 can reverse aberrant tumor lipid metabolism to some extent, remodel the tumor immune microenvironment, and help strengthen the therapeutic outcome of anti-PD-1 immunotherapy. Preliminary analysis of clinical specimens suggests that the USP21-SCD1 axis may serve as a candidate biomarker for predicting immunotherapy response in NSCLC (Fig. 9).

**Figure 9:**
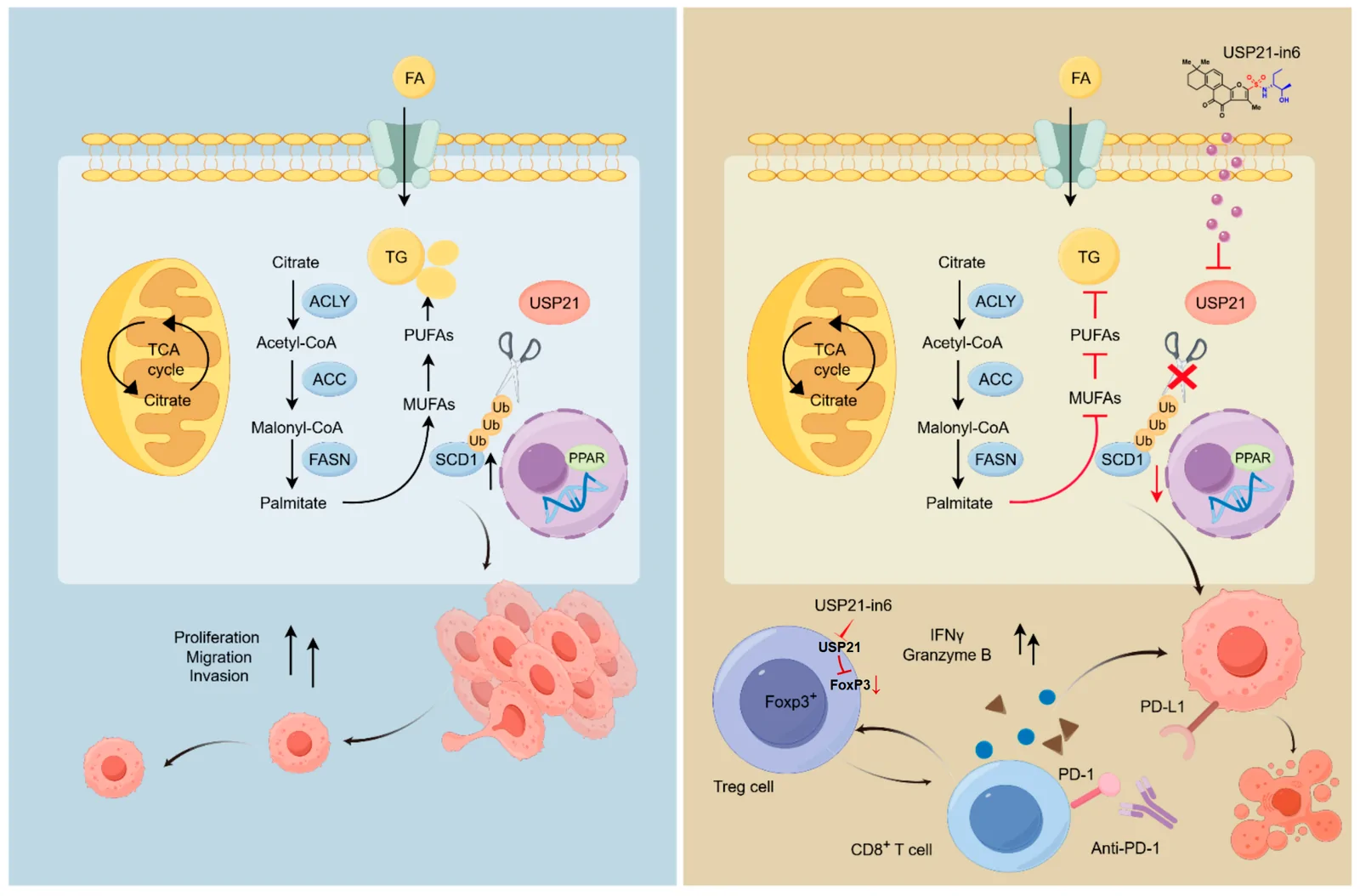
**A model depicting USP21 as an immunometabolic coordinator in NSCLC and its therapeutic targeting.** The deubiquitinase USP21 promotes tumor lipid synthesis by stabilizing SCD1 and maintains immunosuppressive tregs by stabilizing Foxp3. Inhibition by USP21-in6 concurrently disrupts these pathways to suppress tumor growth and enhance anti-tumor immunity, while co-elevated USP21/SCD1 expression correlates with clinical resistance to PD-1 blockade. Abbreviation: FA, fatty acid; TG, triglyceride; TCA, tricarboxylic acid; ACLY, ATP-citrate lyase; ACC, acetyl-CoA carboxylase; FASN, fatty acid synthase; PUFAs, polyunsaturated fatty acids; MUFAs, monounsaturated fatty acids; SCD1, stearoyl-CoA desaturase 1; Ub, ubiquitin; PPAR, peroxisome proliferator-activated receptor; USP21, ubiquitin-specific peptidase 21; IFNγ, interferon-gamma; PD-1, programmed cell death protein 1; PD-L1, programmed death-ligand 1; Foxp3, forkhead box P3; CD8, cluster of differentiation 8.

## Data Availability

All data are available in the main text or the Supplementary Materials.

## Supplementary Materials

The supplementary material is available online at https://www.techscience.com/doi/10.32604/or.2026.089127/s1.
