# Supplementary material for: A Tanshinone IIA Sulfonate Derivative Targets USP21 to Induce SCD1 Degradation, Suppressing NSCLC Metabolism and Enhancing Antitumor Immunity
Source: Oncol Res. 2026 Oct 28;34(11):19. doi: 10.32604/or.2026.089127 (PMC13732340; doi:10.32604/or.2026.089127)
Supplement: Supplementary file 1 [file OncolRes-34-89127-s001.zip › OR-89127-Supplementary_figures.docx]

**Supplementary figures and legends**

**Figure S1.** RMSD profiles of USP21 and potential inhibitors during MD simulations. This plot depicts the RMSD of USP21 (red curve) and the potential inhibitors (blue curve). For clearer visualization, only the first 10 ns segment from the total 50-ns MD simulation trajectory is shown here.

Abbreviations: RMSD, root-mean-square deviation; USP21, ubiquitin-specific peptidase 21.

**Figure S2.** Relevant characterization of Tanshinone IIA and USP21-in6. (A) Chemical structure of Tanshinone IIA, the desulfonated analogue of the lead compound; the structure of the lead compound (Tanshinone IIA Sulfonate, sodium salt) is shown in Figure 1A.. (B) Tanshinone IIA inhibited USP21 enzyme activity. Data are shown as mean ± SD (n=3). (C) Synthetic scheme for the preparation of USP21-in6. (D) ^13^C NMR spectrum of USP21-in6. (E) ¹H NMR spectrum of USP21-in6.Compound 1 (Red powder)

¹H NMR (400 MHz, DMSO‑d₆, 298 K): δ 7.99 (1H, d, J = 8.31 Hz), 7.88 (1H, d, J = 8.13 Hz), 7.64 (1H, d, J = 8.13 Hz), 4.52 (2H, t, J = 5.20 Hz), 3.87–3.77 (1H, m), 3.50–3.45 (1H, m), 3.29–3.24 (1H, m), 3.10 (2H, t, J = 6.24 Hz), 2.43 (3H, s), 1.78–1.70 (2H, m), 1.65–1.60 (2H, m), 1.30 (6H, s), 0.97 (3H, d, J = 6.37 Hz). The signals of active hydroxyl (−OH) protons are overlapped by the broad residual water peak of DMSO‑d₆ at ~3.3–3.5 ppm and cannot be clearly resolved.

¹³C NMR (101 MHz, DMSO‑d₆, 298 K): δ 181.9, 175.3, 160.4, 151.1, 147.1, 143.5, 133.9, 128.0, 126.0, 125.1, 120.9, 119.9, 64.9, 61.1, 60.8, 37.8, 35.0, 31.9 (2C), 30.0, 19.8, 19.2, 9.8.

¹H NMR spectra were acquired on a Bruker Avance 400 spectrometer using the zg30 pulse program (TD = 65536, NS = 16, D1 = 1.00 s, TE = 298 K) in DMSO‑d₆.

¹³C NMR spectra were recorded on a Bruker Avance spectrometer using the zgpg30 pulse program (TD = 32768, NS = 4096, DS = 4, D1 = 2.00 s, D11 = 0.03 s, TE = 298 K) in DMSO‑d₆.

Abbreviations: DUB: Deubiquitinase, DCM, dichloromethane; TEA, triethylamine; PCl₅, phosphorus pentachloride; POCl₃, phosphorus oxychloride; DMF, N,N-dimethylformamide.

**Figure S3.** Selectivity profile for USP21-in6 on a DUB panel comprising 18 individual DUBs. Seventeen DUB dose‑response curves are presented in this figure; the 18th member of the panel, USP21, is shown separately in Figure 1E. Data are presented as mean ± SD (n=3). X‑axis concentrations are in μM and log10‑transformed. Y‑axis represents relative DUB activity normalized to vehicle‑only control (set as 100%). Solid lines are nonlinear‑regression fitting curves. Data are presented as mean ± SD (n=3 biological replicates). All IC50 values shown within panels are in μM.

Abbreviations: MM, molecular mechanics; Asp, aspartic acid; Glu, glutamic acid; Gln, glutamine; Asn, asparagine; Arg, arginine; Phe, phenylalanine; Val, valine; His, histidine; Tyr, tyrosine.

**Figure S4.** Key amino acid residue analysis. (A) Conservation grades were mapped onto the USP21 structure using the ConSurf server. The color code (cyan to purple) corresponds to grades 1 (variable) through 9 (conserved). (B) Per‑residue MM and polar interaction energy contributions for binding‑site residues of the USP21‑in6/USP21 complex. Per‑residue (residue) decomposition quantifies each amino‑acid residue’s contribution to protein‑inhibitor binding free‑energy. Polarity here denotes polar energy components including electrostatic and polar solvation terms calculated by MM‑PBSA. Blue bars represent molecular‑mechanics (MM) energy; red bars represent polar energy. Trajectory sampling: after discarding the initial 10 ns equilibration period, frames were extracted every 100 ps from the remaining 40 ns of the total 50 ns production‑run MD trajectory for MM‑PBSA calculations. Energy values are in kJ·mol^-1^.

Abbreviations: USP21, ubiquitin-specific peptidase 21; USP21-in6: ubiquitin-specific peptidase 21 inhibitor6.

**Figure S5.** USP21-in6 suppresses colony formation and migration of H1299 and H460 cells. (A) Representative colony formation images of H1299 and H460 cells treated with increasing concentrations (0-10 µM) of USP21-in6. Scale bar = 10 mm. (B, C) Quantitative analysis of relative colony numbers in H1299 and H460 cells after USP21‑in6 treatment. Colony numbers were normalized to the vehicle‑treated control group (set as 1). (D) Representative wound-healing assay images of H1299 and H460 cells treated with 0-10 µM USP21-in6, captured at 0 h, 24 h, and 48 h post-wounding. Scale bar = 100 µm. (E, F) Quantitative analysis of relative migration distance in H1299 and H460 cells at 24 h and 48 h after USP21-in6 treatment. For colony formation and wound-healing assays, each USP21-in6 concentration group (0.5–10 µM) was compared with the matched 0 µM vehicle (DMSO) control group, which was set as 1; for wound healing, the 24 h and 48 h time points were additionally compared with the 0 h baseline within each concentration. Data are mean ± SD (n = 3 independent experiments per group). For colony formation (B, C), ordinary one-way ANOVA followed by Dunnett's multiple-comparisons test was performed, comparing each USP21-in6 concentration with the 0 µM vehicle control. For wound-healing assays (E, F), two-way ANOVA (concentration × time) followed by Tukey's multiple-comparisons test was performed. Normality and homogeneity of variance were assessed by the Shapiro–Wilk and Brown–Forsythe tests, respectively, and the assumptions were met. Technical replicates were averaged, and the three independent experiments were treated as biological replicates. * *P* < 0.05; ** *P* < 0.01; ****P* < 0.001, ns *P* ≥ 0.05.

Abbreviations: USP21, ubiquitin-specific peptidase 21; USP21-in6: ubiquitin-specific peptidase 21 inhibitor6.

**Figure S6.** USP21-in6 inhibits cell invasion and induces apoptosis in H1299 and H460 cells. (A) Representative images of Transwell invasion assays for H1299 and H460 cells treated with increasing concentrations (0-10 µM) of USP21-in6 (stained with crystal violet). Scale bar = 100 μm. (B, C) Quantitative analysis of relative invaded cell numbers in H1299 and H460 cells after USP21-in6 treatment. (D) Representative flow cytometry plots (Annexin V-FITC/PI staining) showing apoptosis of H1299 and H460 cells treated with 0-5 µM USP21-in6. The apoptosis rate was defined as the sum of Q2 and Q3 (early plus late apoptotic cells). (E, F) Quantitative analysis of relative apoptosis rates in H1299 and H460 cells after USP21-in6 treatment. For Transwell invasion and apoptosis assays, each USP21-in6 concentration group was compared with the matched 0 µM vehicle (DMSO) control group, and values were normalized to the corresponding 0 µM control (set as 1).. Data are mean ± SD (n = 3 independent experiments per group). Statistical significance was determined using a two-tailed unpaired Student’s t-test or one-way ANOVA. ** *P* < 0.01; ****P* < 0.001, ns *P* ≥ 0.05.

Abbreviation: FITC, fluorescein isothiocyanate; ubiquitin-specific peptidase 21. USP21-in6: ubiquitin-specific peptidase 21 inhibitor6.

**Figure S7.** USP21 knockdown efficiency in H460 and H1299 cells via shRNA and siRNA. (A, B) Representative immunoblot images showing USP21 protein levels in H460 and H1299 cells transfected with negative control shRNA (shNC) or three independent USP21-targeting shRNAs (shUSP21#1, #2, #3). (C, D) Quantitative analysis of USP21/GAPDH protein ratios in shRNA-transfected H460 and H1299 cells. (E, F) Representative immunoblot images showing USP21 protein levels in H460 and H1299 cells transfected with negative control siRNA (siNC) or three independent USP21-targeting siRNAs (siUSP21#1, #2, #3). (G, H) Quantitative analysis of USP21/GAPDH protein ratios in siRNA-transfected H460 and H1299 cells. Protein molecular weights (unit: kDa) are labeled beside each target band. For knockdown-efficiency analysis, each USP21-targeting shRNA (shUSP21#1–3) or siRNA (siUSP21#1–3) group was compared with the matched negative-control group (shNC or siNC, respectively) within the same cell line. Data are mean ± SD (n = 3 independent experiments per group). Statistical significance was determined using a two-tailed unpaired Student’s t-test or one-way ANOVA. * *P* < 0.05; ** *P* < 0.01; ****P* < 0.001, ns *P* ≥ 0.05.

Abbreviation: shNC, short hairpin RNA negative control; shUSP21, short hairpin RNA targeting USP21; siNC, small interfering RNA negative control; siUSP21, small interfering RNA targeting USP21; GAPDH, glyceraldehyde-3-phosphate dehydrogenase; USP21: ubiquitin-specific peptidase 21.

**Figure S8.** USP21 stabilizes SCD1 protein via deubiquitination in H1299 and H460 cells. (A) Representative immunoblot images showing SCD1 protein levels in shNC/shUSP21-transfected H1299 and H460 cells. Cells were treated with chloroquine (CQ, 20 μM, 24 h; lysosomal inhibitor) or MG132(10 μM, 6 h; proteasomal inhibitor) after shRNA transfection. (B, C) Quantitative analysis of relative SCD1 protein expression in H1299 and H460 cells (from panel A). (D) Representative immunoblot images of SCD1 protein stability in shNC/shUSP21-transfected H1299/H460 cells treated with CHX for 0–6 h. (E, F) Quantitative analysis of relative SCD1 protein expression (from panel D) in H1299 and H460 cells. (G) Representative immunoblot images of SCD1 protein stability in H1299/H460 cells treated with 5 µM USP21-in6 and CHX (0–6 h). (H, I) Quantitative analysis of relative SCD1 protein expression (from panel G) in H1299 and H460 cells. (J) Representative immunoblot images showing SCD1 levels in shNC/shUSP21-transfected H1299 cells treated with/without MG132. (K) Representative immunoblot images of SCD1 levels in shNC/shUSP21-transfected H460 cells treated with/without MG132. (L, M) Quantitative analysis of relative SCD1 protein expression (from panels J, K) in H1299 (L) and H460(M) cells. (N) Representative immunoblot images of SCD1/USP21 levels in H1299 cells treated with increasing concentrations of USP21-in6 (0–10 µM) for 24 h, with or without MG132 (10 µM, 6 h; proteasome inhibitor). (O) Quantitative analysis of relative SCD1 protein expression (from panel N) in H1299 cells. (P) Representative immunoblot images of SCD1/USP21 levels in H460 cells treated with increasing concentrations of USP21-in6 (0-10 µM) with/without MG132. (Q) Quantitative analysis of relative SCD1 protein expression (from panel P) in H460 cells. Comparisons were defined as follows: shUSP21 versus shNC (matched control); inhibitor-treated versus untreated within the same knockdown background (MG132 or chloroquine versus vehicle; USP21-in6 each concentration versus 0 µM vehicle); and each CHX chase time point versus the 0 h baseline within the same group. Data are mean ± SD (n = 3 independent experiments per group).. Statistical significance was determined using a two-tailed unpaired Student’s t-test or one-way ANOVA. ** *P* < 0.01; ****P* < 0.001, ns *P* ≥ 0.05.

Abbreviation: GAPDH, glyceraldehyde-3-phosphate dehydrogenase; SCD1, stearoyl-CoA desaturase 1; shNC, short hairpin RNA negative control; shUSP21, short hairpin RNA targeting USP21; CQ, chloroquine; MG132, proteasome inhibitor MG132; CHX, cycloheximide; USP21-in6: ubiquitin-specific peptidase 21 inhibitor6.

**Figure S9.** Semi-quantitative densitometry of linkage-specific SCD1 ubiquitination (related to Fig. 4J). The HA-ubiquitin smear above the SCD1 band in the IP:Flag/IB:HA blot (Fig. 4J) was quantified by densitometry. For each ubiquitin linkage type, the +USP21 value was normalized to the matched-USP21 control (set as 1.0). Data are derived from a single representative experiment and are presented as semi-quantitative.

Abbreviation: USP21, ubiquitin-specific peptidase 21. Myc:Myc epitope tag.

**Figure S10.** Histopathological analysis of mouse major tissues via H&E staining. Representative H&E-stained histological images of tumor, heart, liver, spleen, lung, and kidney tissue sections from mice in different treatment groups.The detailed treatment regimens for each group are listed as follows: (1) Vehicle control (saline containing 5% DMSO and 5% Tween-80); (2) Low-dose USP21-in6 (10 mg/kg, intraperitoneal injection, daily); (3) High-dose USP21-in6 (20 mg/kg, intraperitoneal injection, daily); (4) SCD1 inhibitor (A939572, 10 mg/kg, intraperitoneal injection, daily); (5) Anti-PD-1 antibody (5 mg/kg, intraperitoneal injection, twice a week); (6) Combination treatment of USP21-in6 (10 mg/kg) and A939572 (10 mg/kg); (7) Combination treatment of USP21-in6 (10 mg/kg) and anti-PD-1 antibody (5 mg/kg, twice a week). Scale bar = 100 µm. No obvious histopathological abnormalities were observed in major organs across all treatment groups. We acknowledge that formal blinded scoring or quantitative histopathological assessment was not performed; the H&E analysis was qualitative and intended as a preliminary safety screen. n = 5 mice per group.

Abbreviation: PD-1, programmed death-1. SCD1-in: stearoyl-CoA desaturase-1 inhibitor.
