# Supplementary material for: A Tanshinone IIA Sulfonate Derivative Targets USP21 to Induce SCD1 Degradation, Suppressing NSCLC Metabolism and Enhancing Antitumor Immunity
Source: Oncol Res. 2026 Oct 28;34(11):19. doi: 10.32604/or.2026.089127 (PMC13732340; doi:10.32604/or.2026.089127)
Supplement: Supplementary file 1 [file OncolRes-34-89127-s001.zip › OR-89127-Supplementary_tables.docx]

**Supplementary tables**

**Table S1** Purified Protein Information

| **Name** | **Manufacturer** | **Country** | **City** | **State abbreviation** |  |
| --- | --- | --- | --- | --- | --- |
| USP2 (E-504-050) | R&D Systems | USA | Minneapolis | MN |  |
| USP3 (TP309550) | Origene | USA | Rockville | MD |  |
| USP5 (TP302624) | Origene | USA | Rockville | MD |  |
| USP7 (E-519) | Boston Biochem | USA | Cambridge | MA |  |
| USP9X (BP12796-02) | Biortus | China | Jiangyin | not applicable |  |
| USP10 (E-592) | Boston Biochem | USA | Cambridge | MA |  |
| USP14 (TP300337) | Origene | USA | Rockville | MD |  |
| USP15 (E-594-050) | R&D Systems | USA | Minneapolis | MN |  |
| USP20 (ab269119) | Abcam | UK | Cambridge | not applicable |  |
| USP21 (BP12924-00A) | Biortus | China | Jiangyin | not applicable |  |
| USP21 (E-622) | Boston Biochem | USA | Cambridge | MA |  |
| USP22 (BP12418-01A) | Biortus | China | Jiangyin | not applicable |  |
| USP25 (E-546-050) | R&D Systems | USA | Minneapolis | MN |  |
| USP27 (TP327391) | Origene | USA | Rockville | MD |  |
| USP28 (E-570) | Boston Biochem | USA | Cambridge | MA |  |
| USP37 (BP12420-00A) | Biortus | China | Jiangyin | not applicable |  |
| OTUB1 (BP12800-04A) | Biortus | China | Jiangyin | not applicable |  |
| OTUD5 (BP12777-04A) | Biortus | China | Jiangyin | not applicable |  |
| ATXN3 (E-341) | Boston Biochem | USA | Cambridge | MA |  |

Abbreviations: USP, ubiquitin-specific protease; OTUB1, OTU domain-containing ubiquitin aldehyde-binding protein 1; OTUD5, OTU domain-containing protein 5; ATXN3, ataxin-3; MN, Minnesota; MD, Maryland; MA, Massachusetts.

Table S2 Information of Lentiviruses and Plasmids

| Name | Sequence (5' to 3') |
| --- | --- |
| siRNA for human USP21 | siRNA#1：GGCUACACCUUGAAAUCAATT |
|  | siRNA#2：GCAAGAUUGUGGACCUGUUTT |
|  | siRNA#3：CACUAAGGAAGAAGAGCUATT |
| siRNA for human SCD1 | siRNA：GAAUAUGUCUGGAGAAACAtt |
| shRNA for human USP21 | shRNA#1：CCAGTTGAAAAGTTGTCTCAA |
|  | shRNA#2：TTGTTTCAACCTTTTCACTAA |
|  | shRNA#3：TACCTCAGAGACGTCTATTTT |

Abbreviations: siRNA, small interfering RNA; shRNA, short hairpin RNA.

USP21, ubiquitin-specific peptidase 21;

SCD1, stearoyl-CoA desaturase 1;

**Table S3** Primer Sequence Information

| **Primer Name** | **Primer Sequence(5′→3′)** |
| --- | --- |
| H-ACLY-FO | TCGGCCAAGGCAATTTCAGAG |
| H-ACLY-RE | CGAGCATACTTGAACCGATTCT |
| H-USP21-FO | CAGGTCTGCCTGATGAACGG |
| H-USP21-RE | GCTAAGTTGGTCCGAGATGGG |
| H-ACC1-FO | ATGTCTGGCTTGCACCTAGTA |
| H-ACC1-RE | CCCCAAAGCGAGTAACAAATTCT |
| H-FASN-FO | AAGGACCTGTCTAGGTTTGATGC |
| H-FASN-RE | TGGCTTCATAGGTGACTTCCA |
| H-SCD1-FO | TCTAGCTCCTATACCACCACCA |
| H-SCD1-RE | TCGTCTCCAACTTATCTCCTCC |
| H-ACSL4-FO | TCTGCTTCTGCTGCCCAATT |
| H-ACSL4-RE | CGCCTTCTTGCCAGTCTTTT |
| H-GAPDH-FO | CATG TTCGTCATGGGTGTGAA |
| H-GAPDH-RE | GGCATGGACTGTGGTCATGAG |

Abbreviations: H, Homo sapiens; FO, forward primer; RE, reverse primer.

**Table S4** Antibody Name, Manufacturer, Country of Origin

| **Name** | **Manufacturer** | **Country** | **City** | **State abbreviation** | **Dilution Ratios** |
| --- | --- | --- | --- | --- | --- |
| USP21（17856-1-AP） | Proteintech | China | Wuhan | not applicable | WB:1:1000 |
| USP21（sc-515911） | Santa | USA | Dallas | TX | WB:1:200 |
| USP21（PA5-110556） | Invitrogen | USA | Waltham | MA | WB:1:1000 |
| SCD1（28678-1-AP） | Proteintech | China | Wuhan | not applicable | WB:1:1000 |
| FASN（10624-2-AP） | Proteintech | China | Wuhan | not applicable | WB:1:1000 |
| ACC1（21923-1-AP） | Proteintech | China | Wuhan | not applicable | WB:1:1000 |
| ACLY（15421-1-AP） | Proteintech | China | Wuhan | not applicable | WB:1:1000 |
| ACSL4（22401-1-AP） | Proteintech | China | Wuhan | not applicable | WB:1:1000 |
| β-actin（66009-1-lg） | Proteintech | China | Wuhan | not applicable | WB:1:5000 |
| GAPDH（10494-1-AP） | Proteintech | China | Wuhan | not applicable | WB:1:5000 |
| HA-tag（3724S） | Cell Signaling Technology | USA | Danvers | MA | WB:1:1000 |
| Myc-tag（16286-1-AP） | Proteintech | China | Wuhan | not applicable | WB:1:1000 |
| Flag-tag（66008-4-Ig） | Proteintech | China | Wuhan | not applicable | WB:1:2000 |
| panCK (26411‑1‑AP) | Proteintech | China | Wuhan | not applicable | IHC:1:200; |
| Alexa Fluor® 700 anti-mouse CD45（147716） | Biolegend | USA | San Diego | CA | Flow cytometry:1:200 |
| FITC Rat Anti-Mouse CD4(RM4-5)（553046） | BD Pharmingen | USA | San Jose | CA | Flow cytometry:1:200 |
| BV510 Rat Anti-Mouse CD8a(53-6.7)（563068） | BD Pharmingen | USA | San Jose | CA | Flow cytometry:1:200 |
| FOXP3 Monoclonal Antibody (FJK-16s), APC, eBioscience™（17-5773-82） | Thermo | USA | San Diego | CA | Flow cytometry:0.5 μg/test |
| PE-Cy7 Rat Anti-Mouse IFN-γ(XMG1.2)（557649） | BD Pharmingen | USA | San Diego | CA | Flow cytometry:0.5 μg/test |
| PE anti-human/mouse Granzyme B Recombinant（372208） | Biolegend | USA | San Jose | CA | Flow cytometry:1:200 |
| PE-Cy7 Rat Anti-Mouse CD25(PC61)（552880） | BD Pharmingen | USA | San Diego | CA | Flow cytometry:1:200 |

Abbreviations: WB, Western blotting; IHC, immunohistochemistry; APC, allophycocyanin; FITC, fluorescein isothiocyanate; BV510, Brilliant Violet 510; PE, phycoerythrin; Cy7, cyanine 7; CD, cluster of differentiation; IFN-γ, interferon-γ; FoxP3, forkhead box P3; HA, hemagglutinin; panCK, pan-cytokeratin.

FASN, fatty acid synthase; USP21, ubiquitin-specific peptidase 21; SCD1, stearoyl-CoA desaturase 1; ACLY, ATP citrate lyase; ACSL4, acyl-CoA synthetase long-chain family member 4; ACC1, acetyl-CoA carboxylase 1; GAPDH, glyceraldehyde‑3‑phosphate dehydrogenase; Myc, Myc epitope tag; Flag, FLAG epitope tag; CA, California; MA, Massachusetts. BD, Becton, Dickinson and Company; TX, Texas; CA, California; MA, Massachusetts; FJK-16s and XMG1.2, monoclonal antibody clone designations.

**Table S5** Chemical Reagents Name, Manufacturer, Country of Origin

| **Name** | **Catalog number** | **Manufacturer** | **Country** | **City** | **State abbreviation** |
| --- | --- | --- | --- | --- | --- |
| DMEM medium | MA0212 | Meilunbio | China | Dalian | not applicable |
| RPMI 1640 medium | MA0215 | Meilunbio | China | Dalian | not applicable |
| Fetal Bovine Serum (FBS) | FSP500 | Excell | China | Nanjing | not applicable |
| Penicillin-Streptomycin solution | G4003 | Servicebio | China | Wuhan | not applicable |
| Trypsin-EDTA (0.02% EDTA) | G4012 | Servicebio | China | Wuhan | not applicable |
| Plasmid Extraction Kit | D6942-01 | Omega Bio-tek | USA | GA | Norcross |
| Lipofectamine 2000 | 11668019 | Invitrogen | USA | Waltham | MA |
| Protein Marker / Protein Ladder | 26616 | Thermo Fisher | USA | Waltham | MA |
| Cross-linked Sepharose CL-2B | S8731 | Solarbio | China | Beijing | not applicable |
| MG132 (Proteasome inhibitor) | HY-13259 | MCE | USA | Monmouth Junction | NJ |
| Chloroquine | C805324 | Macklin | China | Shanghai | not applicable |
| Dimethyl sulfoxide (DMSO) | D2650 | Sigma | USA | St. Louis | MO |
| TE buffer | ST725 | Beyotime | China | Shanghai | not applicable |
| BCA Protein Assay Kit | P0012 | Beyotime | China | Shanghai | not applicable |
| Cycloheximide (CHX) | U1013 | UNIV-BIO | China | Shanghai | not applicable |
| PCR Kit | Q711-02 | Vazyme | China | Nanjing | not applicable |
| Reverse Transcription Kit | R223-01 | Vazyme | China | Nanjing | not applicable |
| Paraformaldehyde (PFA) | P8045 | Solarbio | China | Beijing | not applicable |
| Protein G Sepharose 4 FF | 17-0618-01 | Cytiva (formerly GE Healthcare Life Sciences)Swedennot applicable | Sweden | Uppsala | not applicable |
| PBS powder | P1010 | Solarbio | China | Beijing | not applicable |
| Triton X-100 | T8200 | Solarbio | China | Beijing | not applicable |
| Puromycin | MB2771 | Meilunbio | China |  |  |
| TBS powder | T1082 | Solarbio | China | Beijing | not applicable |
| Tween 20 | T8220 | Solarbio | China | Beijing | not applicable |
| LB agar powder | L1015 | Solarbio | China | Beijing | not applicable |
| Red Blood Cell Lysis Buffer | abs9101 | Absin | China | Shanghai | not applicable |
| Free Fatty Acid Assay Kit | BC0595 | Solarbio | China | Beijing | not applicable |
| Oil Red O Staining Kit | G1262 | Solarbio | China | Beijing | not applicable |
| Apoptosis Assay Kit | KTA0002 | Abbkine | China | Wuhan | not applicable |
| Hexane | No.110-54-3 | Kermel | China | Tianjin | not applicable |
| Di-Ub | P20022 | Solarbio | China | Beijing | not applicable |
| CCK-8 | MA0218 | Meilunbio | China | Dalian | not applicable |
| Matrigel | 082704 | Mogengel Biotech | China | Shanghai | not applicable |
| BODIPY 493/503 (D3922) | D3922 | Invitrogen | USA | Waltham | MA |
| SCD1 inhibitor | A939572 | Bide Pharmatech | China | Shanghai | not applicable |
| PD-1 antibody | P372 | Leinco | USA | Fenton | MO |
| ALT Assay Kit | BC1555 | Solarbio | China | Beijing | not applicable |
| AST Assay Kit | BC1565 | Solarbio | China | Beijing | not applicable |
| LIVE/DEAD Viability/Cytotoxicity Kit | L3224 | Thermo Fisher | USA | Waltham | MA |
| Collagenase IV (LS004188) | LS004188 | Worthington | USA | Lakewood | NJ |
| DNase I | D8071 | Solarbio | China | Beijing | not applicable |
| Tribromoethanol | T48402 | Sigma | USA | St. Louis | MO |
| Mouse BD Fc Block | 553141 | BD Pharmingen | USA | USSan DiegoA | CA |
| ULTRAcomp eBeads Compensation Beads | 01-2222-42 | Thermo Fisher | USA | Waltham | MA |
| Cell Freezing Medium | BL203B | Biosharp | China | Beijing | not applicable |

Abbreviations: DMEM, Dulbecco's Modified Eagle Medium; FBS, fetal bovine serum; EDTA, ethylenediaminetetraacetic acid; PFA, paraformaldehyde; PBS, phosphate-buffered saline; TBS, Tris-buffered saline; TE, Tris-EDTA buffer; BCA, bicinchoninic acid; CHX, cycloheximide; DMSO, dimethyl sulfoxide; PCR, polymerase chain reaction; CCK-8, Cell Counting Kit-8; BODIPY, boron-dipyrromethene; DNase I, deoxyribonuclease I; SCD1, stearoyl‑CoA desaturase 1; PD-1, programmed death-1; ALT, alanine aminotransferase; AST, aspartate aminotransferase; RPMI, Roswell Park Memorial Institute medium; CL, cross-linked; PCR, polymerase chain reaction; LB, lysogeny broth; Di-Ub, di-ubiquitin; IV, intravenous; Fc, crystallizable fragment. LIVE/DEAD and ULTRAcomp eBeads are commercial product names.

**Table S6** Name, manufacturer and country of experimental-use instruments

| **Name** | **Manufacturer** | **Model** | **Country** | **City** | **State abbreviation** |
| --- | --- | --- | --- | --- | --- |
| Basic electrophoresis equipment | Bio‑Rad | PowerPac Basic | USA | Hercules | CA |
| Basic PCR instrument | Bio‑Rad | T100 | USA | Hercules | CA |
| Real‑time PCR system | Bio‑Rad | CFX96 | USA | Hercules | CA |
| −80 °C ultra-low-temperature freezer | Thermo Scientific | Forma 995 | USA | Waltham | MA |
| Dry‑heat sterilizing oven | Thermo Scientific | Heratherm OGS60 | USA | Waltham | MA |
| Constant‑temperature shaker | Thermo Scientific | MaxQ 4000 | USA | Waltham | MA |
| Clean bench | Thermo Scientific | 1300 Series A2 | USA | Waltham | MA |
| Constant‑temperature water bath | Pudong | HWS-26 | China | Shanghai | not applicable |
| Horizontal decoloring shaker | Jingmai | TS-100 | China | Shanghai | not applicable |
| Electrophoresis tank | Liuyi (Beijing) | DYCP-31DN | China | Beijing | not applicable |
| Constant‑temperature incubator | Yiheng | DHP-9162 | China | Shanghai | not applicable |
| 4 °C refrigerator | Haier | HYC-310S | China | Qingdao | not applicable |
| Multimode microplate reader | Thermo Scientific | Varioskan LUX | USA | Waltham | MA |
| −20 °C refrigerator | Haier | DW-40L188 | China | Qingdao | not applicable |
| High‑speed refrigerated centrifuge | Eppendorf | 5424R | Germany | Hamburg | not applicable |
| Micro‑pipette | Eppendorf | Research plus | Germany | Hamburg | not applicable |
| CO₂ incubator | Thermo Scientific | Forma 3111 | USA | Waltham | MA |
| Electronic analytical balance | Sartorius | Quintix 224-1CN | Germany | Göttingen | not applicable |
| Gel imaging system | GE | Amersham Imager 600 | USA | Boston | MA |
| Flow cytometer | Sony Biotechnology | Sony MA900 | USA | San Jose | CA |
| Inverted microscope | Nikon | Eclipse Ti2-U | Japan | Tokyo | not applicable |
| Ultrapure-water system | Millipore | Milli-Q Integral 3 | USA | Burlington | MA |
| Semi-dry transfer cell | Bio-Rad | Trans-Blot SD | USA | Hercules | CA |
| Bio-layer interferometer | ForteBio (Sartorius) | Octet R8 | USA | Fremont | CA |
| Slide scanner (IHC) | 3DHISTECH | Panoramic MIDI | Hungary | Budapest | not applicable |
| Multiplex imaging system (mIF) | Akoya Biosciences | Vectra Polaris | USA | Marlborough | MA |
| Transmission electron microscope | Hitachi | HT7800 | Japan | Tokyo | not applicable |
| GC-MS system | Agilent | 8890 GC + 7000D | USA | Santa Clara | CA |
| NMR spectrometer | Bruker | Avance 400 | USA | Billerica | MA |
| UV-Vis spectrophotometer | Thermo Fisher Scientific | NanoDrop One | USA | Waltham | MA |

Abbreviations: PCR, polymerase chain reaction; CO₂, carbon dioxide; IHC, immunohistochemistry; mIF, multiplex immunofluorescence; GC-MS, gas chromatography–mass spectrometry; NMR, nuclear magnetic resonance; UV-Vis, ultraviolet–visible spectrophotometry; GE, General Electric; CA, California; MA, Massachusetts; USA, United States of America.

**Table S7** Structures, energy calculation results and inhibitory percentages of potential inhibitors.

| **Compound** | **Structure** | **Docking Score (kcal/mol)** | **MM/PBSA Binding Energy(kJ/mol)** | **USP21 inhibition(%) at 20 μM** |
| --- | --- | --- | --- | --- |
| Duvelisib |  | -11.1 | -184.49 | 0.00% |
| Eltrombopag olamine |  | -10.1 | -160.06 | 26.30% |
| Tanshinone IIA Sulfonate |  | -10.6 | -143.71 | 98.00% |
| Quercitrin |  | -10.0 | -158.23 | 30.10% |
| CP21R7 |  | -9.9 | -141.20 | 0.00% |
| Olprinone |  | -9.9 | -109.00 | 0.00% |
| Olaparib |  | -9.8 | -124.12 | 0.00% |
| Lumacaftor |  | -9.8 | -134.09 | 0.00% |
| Ruxolitinib |  | -9.6 | -143.00 | 0.00% |
| Ezetimibe |  | -9.5 | -129.22 | 0.00% |

Note: Top‑ranked 10 compounds are listed, including their docking scores, MM/PBSA binding energy results, and inhibitory activities against USP21. All selected compounds satisfied the screening criterion of ligand RMSD < 3.0 Å during the full‑length 50 ns molecular dynamics simulations; time‑dependent ligand‑RMSD curves (first 10 ns trajectories) are provided in Figure S1. The red font emphasize that the compound, Tanshinone IIA Sulfonate, shows an inhibition rate exceeding 50% against USP21.

Abbreviations: USP21, ubiquitin-specific peptidase 21; MM/PBSA, Molecular Mechanics/Poisson–Boltzmann Surface Area; MD, molecular dynamics; RMSD, root-mean-square deviation; ns, nanosecond; Å, ångström; μM, micromolar; kcal/mol, kilocalories per mole; kJ/mol, kilojoules per mole.

**Table S8** Binding Free Energies of Sodium Tanshinone IIA Sulfonate and USP21-in6 to USP21 Model

| **Compound** | **△EvdW** | **△Eele** | **△GPB** | **△GSA** | **△Gbinding** |
| --- | --- | --- | --- | --- | --- |
| USP21-in6 | -195.61 | -133.70 | 189.91 | -18.13 | -157.53 |
| Tanshinone IIA Sulfonate | -181.71 | -68.83 | 132.33 | -17.76 | -135.97 |

Note: ΔG_binding = ΔEvdW + ΔEele + ΔGPB + ΔGSA. ΔEint cancels out after subtraction among complex, receptor and ligand and is omitted. A total of 100 snapshots were extracted at an interval of 0.5 ns from the last 50 ns of MD simulations and submitted to MMPBSA.py for free‑energy calculation. All energy values are given in kJ/mol.

Abbreviations: ΔEvdW, change in van der Waals energy; ΔEele, change in electrostatic energy; ΔGPB, change in polar solvation energy calculated by the Poisson–Boltzmann method; ΔGSA, change in nonpolar solvation energy (solvent-accessible surface area); ΔGbinding, binding free energy; ΔEint, change in internal energy; MD, molecular dynamics; MM-PBSA, Molecular Mechanics/Poisson–Boltzmann Surface Area. USP21, ubiquitin-specific peptidase 21. USP21-in6: ubiquitin-specific peptidase 21 inhibitor6.

**Table S9** SCD1 ubiquitination sites and supporting evidence number predicted by online tools

| **UniProt** | **Evidence** | **Phosphositeplus** | **Evidence** |
| --- | --- | --- | --- |
| K34 | 21 | K34 | 5 |
| K51 | 28 | K51 | 5 |
| K60 | 21 | K60 | 5 |
| K62 | 7 | K62 | 3 |
| K68 | 10 | K68 | 5 |
| K162 | 6 | K162 | 29 |
| K194 | 5 | K189 | 1 |
| K196 | 25 | K194 | 2 |
| K209 | 20 | K196 | 18 |
| K278 | 1 | K209 | 16 |
| K338 | 5 | K278 | 1 |
| K341 | 12 | K338 | 6 |
| K357 | 9 | K341 | 19 |
|  |  | K357 | 2 |

Note: Ubiquitination‑site information was retrieved from UniProt (https://www.uniprot.org/) and PhosphoSitePlus (https://www.phosphosite.org/), accessed on November 4, 2025. The human canonical SCD1 isoform was used for query without additional custom search criteria. Evidence counts represent the number of independent published experimental records supporting ubiquitination modification at each lysine residue. Sites marked in red were selected to construct lysine‑to‑arginine (K‑to‑R) mutants for subsequent experimental validation.

Abbreviations: SCD1, stearoyl-CoA desaturase 1; K, lysine residue, R, arginine residue; K-to-R, lysine-to-arginine substitution.

**Table S10** Basic and clinical characteristics of NSCLC patients

| **No.** | **Age** | **Gender** | **Tumor Stage** | **Histopathological Type** | **ICB Regimen** | **Response to ICB** |
| --- | --- | --- | --- | --- | --- | --- |
| 1 | 61 | Male | T2bN3M1 | Lung adenocarcinoma | Sintilimab 200 mg iv q3w | No |
| 2 | 58 | Female | T1aN1M1 | Lung adenocarcinoma | Camrelizumab 200 mg iv q3w | No |
| 3 | 69 | Male | T2N0M1 | Lung Squamous Cell Carcinoma | Sintilimab 200 mg iv q3w | No |
| 4 | 69 | Male | T3N2M1 | Lung Squamous Cell Carcinoma | Pembrolizumab 200 mg iv q3w | Yes |
| 5 | 66 | Male | T2N3M0 | Lung Squamous Cell Carcinoma | Camrelizumab 200 mg iv q3w | Yes |
| 6 | 60 | Male | T3N2M0 | Lung adenocarcinoma | Pembrolizumab 200 mg iv q3w | Yes |

Note: Tumor response to ICB therapy was evaluated according to the Response Evaluation Criteria in Solid Tumors version 1.1 (RECIST 1.1). Contrast‑enhanced chest and abdominal CT scans were performed for radiological assessment every two cycles after initiation of PD‑1 inhibitor monotherapy, followed by a 6‑month follow‑up. Responders (“Yes”) were defined as patients achieving complete response (CR) or partial response (PR); non‑responders (“No”) were defined as patients with stable disease (SD) or progressive disease (PD). All agents were administered at standard recommended doses in accordance with clinical guidelines for non‑small‑cell lung cancer. iv, intravenously; q3w, every 3 weeks.

Abbreviations: NSCLC, non-small cell lung cancer; ICB, immune checkpoint blockade; CR, complete response; PR, partial response; SD, stable disease; PD, progressive disease; CT, computed tomography; PD-1, programmed death-1; TNM, tumor–node–metastasis staging system (T, primary tumor; N, regional lymph nodes; M, distant metastasis).
